# Prognostic Value of Tumor Budding and Worst Pattern of Invasion in Clinically Node-Negative Early-Stage Lip and Oral Cavity Squamous Cell Carcinoma: A Systematic Review

**DOI:** 10.3390/jcm15207949

**Published:** 2026-10-14

**Authors:** Mădălina Anca Moldovan, Laura-Veronica Filip, Cristian Niky Cumpată, Bogdan Andrei Bumbu, Paolo Di Francesco, Călin Rareș Roman

**Affiliations:** 1 Department of Oro-Maxillo-Facial Surgery and Implantology, Faculty of Dental Medicine, Iuliu Hațieganu University, 400012 Cluj-Napoca, Romania; madalina.lazar@umfcluj.ro (M.A.M.); raresroman25@gmail.com (C.R.R.); 2 Private Practice, Filip Laura-Veronica, 400584 Cluj-Napoca, Romania; 3 Department of Oral and Maxillofacial Surgery, Faculty of Dental Medicine, Titu Maiorescu University, 031593 Bucharest, Romania; 4 Department of Dental Medicine, University of Oradea, 410073 Bihor, Romania; bogdanbumbu@yahoo.com; 5 Department of Prosthodontics, Faculty of Dental Medicine, Titu Maiorescu University, 031593 Bucharest, Romania; dr.difrancescopaolo@gmail.com

**Keywords:** lip and oral cavity squamous cell carcinoma, tumor budding, worst pattern of invasion, occult cervical metastasis, locoregional recurrence, prognostic markers, systematic review

## Abstract

**Background:** Early-stage squamous cell carcinoma of the lip and oral cavity may behave aggressively despite clinically negative cervical lymph nodes. Tumor budding (TB) and worst pattern of invasion (WPOI) have been proposed as histopathological markers of invasive behavior, but their prognostic value in cT1–2N0M0 disease remains uncertain. **Methods:** This PRISMA 2020 systematic review included studies published as eligible full-text journal articles up to 25 April 2026 and was informed by REMARK and guidance for systematic reviews of prognostic factor studies. PubMed and Scopus were searched as bibliographic databases, supplemented by ScienceDirect, Semantic Scholar, and backward and forward citation searching. Eligible studies assessed TB and/or WPOI in resection specimens from surgically treated cT1–2N0M0 lip/oral cavity squamous cell carcinoma and reported nodal, recurrence, or eligible survival outcomes. Risk of bias was evaluated using the Quality In Prognosis Studies (QUIPS) tool. **Results:** Thirty-eight reports met eligibility criteria; linked or partially overlapping cohorts were treated as related evidence to avoid double counting. Higher-risk WPOI and high-grade TB were associated with cervical nodal disease, locoregional recurrence, and adverse survival across multiple reports, although adjusted effects varied. Eligible survival outcomes included disease-free survival, recurrence-free survival, disease-specific/cancer-specific survival, and overall survival; progression-free survival was excluded. Conclusions: TB and WPOI may provide complementary postoperative prognostic information in clinically node-negative early-stage lip and oral cavity squamous cell carcinoma. Heterogeneity in definitions, thresholds, cervical management, endpoints, and statistical adjustment precluded a single pooled estimate and limits immediate clinical implementation. Neither marker should currently be used as an isolated determinant of treatment decisions.

## 1. Introduction

Lip and oral cavity cancer shows marked geographic, sex-related, and age-related variation in incidence and outcome. According to GLOBOCAN 2024, an estimated 452,205 new cases and 194,108 deaths occurred worldwide, corresponding to age-standardized incidence and mortality rates of 4.5 and 1.9 per 100,000, respectively [1]. Squamous cell carcinoma accounts for approximately 90% of oral malignancies and occurs more frequently in men, particularly in middle-aged and older adults. Its etiology is multifactorial, with tobacco use and chronic alcohol consumption representing major established risk factors, while additional contributors, including betel-quid/areca-nut exposure and other environmental, host-related, and molecular factors, vary across geographic populations [2]. Although oncogenic human papillomavirus (HPV), particularly HPV-16, is most strongly associated with oropharyngeal squamous cell carcinoma, oral HPV infection and HPV-positive tumors are also encountered in the oral cavity, although their etiological significance remains less clearly established. Persistence of oral HPV infection may be influenced by cofactors including tobacco exposure, impaired oral health, and immunosuppression [3].

Although patients with cT1–2N0M0 disease are generally considered to have early-stage disease and a relatively favorable prognosis, clinical outcomes remain heterogeneous. A proportion of clinically node-negative patients develop occult or delayed cervical lymph node metastasis, locoregional recurrence, or disease-related death despite apparently adequate primary treatment [4,5].

Cervical nodal status is one of the most important prognostic determinants in squamous cell carcinoma of the lip and oral cavity (collectively referred to as OSCC in this review). However, clinical examination and conventional imaging cannot reliably identify all microscopic nodal metastases. Consequently, management of the clinically node-negative neck remains challenging, particularly when the estimated risk of occult disease is close to the threshold used to justify elective neck treatment. Conventional pathological parameters, including depth of invasion, tumor differentiation, perineural invasion, lymphovascular invasion, and resection margin status, contribute to postoperative risk assessment but do not fully explain the biological variability observed among tumors of the same clinical stage [4,5].

Tumor budding (TB) and worst pattern of invasion (WPOI) are histopathological features of the invasive tumor front that may provide additional prognostic information. TB is generally defined as isolated tumor cells or small clusters composed of fewer than five cells at the invasive front and is considered a morphological manifestation of reduced cellular cohesion and invasive behavior [6,7]. WPOI describes the most aggressive architectural pattern identified at the tumor–host interface. In the commonly used five-tier classification, WPOI-1 to WPOI-3 represent progressively less cohesive but relatively organized invasion patterns; WPOI-4 consists of small tumor islands, strands, or individual cells; and WPOI-5 is characterized by satellite tumor deposits separated from the primary tumor or the nearest satellite by at least 1 mm [8].

Previous systematic reviews and meta-analyses have linked high-grade TB and non-cohesive WPOI with cervical nodal metastasis, recurrence, and reduced survival in OSCC [4,5,6,7,9,10]. In a comprehensive meta-analysis of head and neck squamous cell carcinoma, Silva et al. reported that high TB was associated with poorer overall survival, disease-free survival, and disease-specific survival, as well as with a higher likelihood of lymph node metastasis [11]. At the individual-study level, Mascitti et al. evaluated alternative TB thresholds in 211 oral tongue squamous cell carcinomas and found that a five-bud cut-off significantly discriminated disease-specific survival [12]. Broader syntheses and methodological studies likewise highlight heterogeneity in prognostic effect estimates, tumor-budding assessment, and reproducibility [13,14,15,16,17,18,19,20]. These findings reinforce the broader prognostic relevance of TB, while WPOI-focused analyses likewise support an adverse association for non-cohesive invasion patterns [4,5,10,13]. However, prior syntheses and primary cohorts frequently included heterogeneous tumor stages, anatomical subsites, nodal status, treatment strategies, and histopathological assessment methods. Their findings therefore cannot be applied directly to surgically treated cT1–2N0M0 OSCC without stage- and subgroup-specific evaluation.

The primary objective of this systematic review was to evaluate the prognostic value of TB and WPOI, assessed in surgical resection specimens, in adults with surgically treated cT1–2N0M0 OSCC. Eligible clinical outcomes included occult or pathological cervical nodal disease, delayed cervical metastasis, and local, regional, or locoregional recurrence. Disease-free survival (DFS), recurrence-free survival (RFS), disease-specific/cancer-specific survival (DSS/CSS), and overall survival (OS) were included as secondary prognostic outcomes when reported. Additional objectives were to describe the assessment methods and thresholds used for TB and WPOI, the AJCC/UICC TNM editions used by the primary studies, the cervical treatment strategies and the methodological quality of the available evidence.

## 2. Materials and Methods

### 2.1. Study Design and Reporting

This systematic review evaluated the prognostic value of TB and WPOI in clinically node-negative, early-stage OSCC. The review was reported in accordance with the Preferred Reporting Items for Systematic Reviews and Meta-Analyses (PRISMA 2020) statement and was informed by the REporting recommendations for tumor MARKer prognostic studies (REMARK) and guidance for systematic reviews of prognostic factor studies [21,22,23,24]. The review question and eligibility criteria were structured according to the PICOTS (Population, Index prognostic factor, Comparator prognostic factor, Outcome, Timing, and Setting) framework, considering the target population, prognostic factors, clinical outcomes, timing of assessment, and study setting.

The review was not prospectively registered in PROSPERO, and no separate review protocol document was prepared. Prospective registration was not completed before study screening commenced; this is acknowledged as a limitation of the review process.

### 2.2. Information Sources and Search Strategy

The literature-search eligibility cutoff was 25 April 2026. As a completeness and reproducibility check, the searches were rerun on 19 August 2026 after removal of the lower publication-date restriction, and an additional Scopus search was performed on 20 August 2026. The eligibility cutoff was not extended; reports published online or in print as eligible full-text journal articles after 25 April 2026 were therefore outside the scope of the review.

PubMed and Scopus were treated as bibliographic databases. ScienceDirect (Elsevier, Amsterdam, The Netherlands), a publisher-hosted full-text discovery platform, and Semantic Scholar, a scholarly literature discovery and citation platform, were retained as supplementary electronic search platforms and were not described as conventional bibliographic databases. The electronic searches were supplemented by backward and forward citation searching using Citationchaser version 0.0.4 [25].

Search strategies combined terms for oral cavity or head and neck squamous cell carcinoma with terms describing TB and WPOI. Outcome terms were not mandatory search components because potentially eligible prognostic studies could report cervical nodal disease, recurrence, DFS, RFS, DSS/CSS, OS, or other oncological outcomes without using uniform outcome terminology in titles or abstracts. The complete search strings, date handling, source classification, and citation-search procedures are provided in Tables S1–S4 of the Supplementary Materials.

### 2.3. Eligibility Criteria

Studies were eligible when they met all the following criteria:

- Included adults aged 18 years or older with primary cT1–2N0M0 OSCC of the lip or oral cavity. Studies containing mixed tumor stages or mixed oral/head-and-neck subsites were eligible only when data for the cT1–2N0M0 lip/oral cavity subgroup could be extracted separately.
- Reported cT1–2N0M0 disease or stage I–II disease according to the staging system used in the original report, with sufficient clinical information to confirm eligibility. No lower publication-date limit or restriction by TNM edition was applied; the AJCC/UICC TNM edition used by each report was recorded when stated.
- Included patients treated surgically with curative intent, with or without elective neck dissection or sentinel lymph node biopsy.
- Assessed TB and/or WPOI in surgical resection specimens.
- Used an established or clearly described histopathological assessment system for TB and/or WPOI, including a defined TB counting or scoring method and/or analytical threshold, the Brandwein–Gensler WPOI classification, the CAP framework, or a compatible five-tier WPOI system [8,26,27].
- Investigated the association between TB and/or WPOI and at least one eligible outcome: occult or pathological cervical nodal involvement, sentinel lymph node biopsy positivity, delayed cervical metastasis, local recurrence, regional recurrence, locoregional recurrence, DFS, RFS, DSS, CSS, or OS.
- Were observational studies, including cohort or cross-sectional designs.
- Were available as full-text journal articles written in English.

Studies were excluded if they:

- Included non-squamous histological subtypes or potentially malignant oral lesions;
- Included tumors outside the lip and oral cavity or mixed stages/subsites without separately extractable data for the eligible cT1–2N0M0 lip/oral cavity population;
- Evaluated patients who had received neoadjuvant chemotherapy or radiotherapy;
- Did not include primary surgical treatment;
- Assessed TB or WPOI exclusively in incisional or diagnostic biopsy specimens;
- Did not provide an eligible TB/WPOI assessment for the extractable cT1–2N0M0 cohort or subgroup;
- Did not report an association between the investigated marker and an eligible nodal, recurrence, or survival outcome;
- Used a novel or insufficiently described scoring system that did not permit an interpretable assessment of TB or WPOI;
- Were reviews, meta-analyses, case reports, experimental studies, animal studies, or molecular/genetic investigations without an eligible clinical cohort.

### 2.4. Study Selection and Report Retrieval

Records retrieved from all sources were consolidated in a master dataset. Mendeley [28] and Microsoft Excel [29] were used to organize references and support deduplication, screening, and eligibility assessment. Duplicate records were identified using bibliographic identifiers, particularly DOI when available, together with conservative title, author, and publication-year matching.

Following cross-source deduplication, all unique records (*n* = 1831) entered title and abstract screening. Artificial-intelligence-assisted support (ChatGPT, GPT-5.6, OpenAI, San Francisco, CA, USA) was not used to pre-filter, rank, or exclude records from the human screening set and did not make autonomous eligibility decisions. AI assistance was limited to organization of screening records, bibliographic cross-checking, flagging potentially relevant or potentially inconsistent records for additional human review, and consistency checks across extracted data and manuscript tables.

Two reviewers independently screened the complete set of 1831 titles and abstracts against the eligibility criteria and subsequently independently assessed the full texts of potentially eligible reports. Disagreements or uncertainties were resolved through discussion; when consensus could not be reached, a third reviewer was consulted. Reasons for exclusion at the full-text stage were recorded during full-text screening. All final eligibility decisions were made by the reviewers and verified against the original reports. All reports selected for full-text assessment were successfully retrieved and assessed for eligibility.

For the final extraction, study-level data entering the synthesis were checked directly against primary reports. When a numerical effect estimate could not be verified directly in the primary report, the association was summarized qualitatively, and no numerical estimate from a secondary extraction was substituted.

### 2.5. Data Extraction and Management of Overlapping Cohorts

Data were extracted using a standardized framework covering study design and population characteristics, clinical staging and tumor site, the AJCC/UICC TNM edition used, cervical assessment and treatment, TB and WPOI definitions and evaluation methods, additional histopathological variables, follow-up and adjuvant therapy, relevant oncological outcomes, and the statistical associations between TB or WPOI and those outcomes. Modified or study-specific WPOI definitions were recorded explicitly and were not assumed to be interchangeable with the conventional five-tier framework. In particular, the expanded WPOI-5 construct used in the linked Cork reports incorporated extratumoral PNI and/or LVI in addition to satellite tumor nodules separated by at least 1 mm; these reports were therefore interpreted separately from studies using the conventional satellite-based WPOI-5 definition.

For each report–outcome pair, effect estimates were extracted when available, including odds ratios, risk ratios, or hazard ratios with 95% confidence intervals; univariable and adjusted estimates were distinguished. When a primary report provided a time-to-event analysis, the reported survival estimate or hazard ratio was preferred over a newly calculated crude effect estimate from final event counts.

Reports originating from the same or partially overlapping patient cohort were identified using the institution, recruitment period, sample characteristics, and author group. Linked reports were retained when they contributed distinct eligible outcomes or analyses, but overlapping participants were not double counted in combined descriptive summaries or any comparative synthesis. Data were extracted by one reviewer and independently verified by a second reviewer.

### 2.6. Outcomes

Clinically distinct outcomes were retained as separate endpoints in the synthesis rather than being treated as interchangeable evidence of a single adverse prognostic outcome. These comprised:

- Occult or pathological cervical nodal disease identified through initial or elective pathological neck assessment;
- Sentinel lymph node biopsy positivity;
- Delayed cervical lymph node metastasis during postoperative surveillance;
- Local, regional, or locoregional recurrence after primary surgical treatment.

Secondary prognostic outcomes included DFS, RFS, DSS, CSS, and OS. Pathological nodal stage and associations between TB or WPOI and other adverse histopathological characteristics were extracted as additional secondary or exploratory associations when reported. DSS and CSS were considered within the same disease-specific mortality domain while preserving the terminology used by the original reports; DFS and RFS were retained separately when their definitions differed. Progression-free survival (PFS) was not considered an eligible survival outcome and, when reported alongside eligible endpoints, was not synthesized as part of the review. Because cervical management differed among primary studies, outcomes identified by elective neck dissection, sentinel lymph node biopsy, and postoperative surveillance were synthesized separately.

### 2.7. Risk-of-Bias Assessment

The risk of bias of the included reports was assessed using the Quality In Prognosis Studies (QUIPS) tool [30,31]. The following six domains were assessed: study participation, study attrition, prognostic factor measurement, outcome measurement, study confounding, and statistical analysis and reporting. Each domain was classified as having a low, moderate, or high risk of bias based on participant selection, completeness of follow-up, definition and reproducibility of the prognostic factor, reliability of outcome measurement, control of relevant confounders, and appropriateness of the statistical analysis. Domain-level judgments were based on the overall pattern and likely impact of item-level concerns within each QUIPS domain rather than on a numerical score; missing or unclear methodological information was not assumed to indicate low risk of bias. Risk-of-bias assessments were performed independently by two reviewers, with disagreements resolved through discussion and, when necessary, consultation with a third reviewer. Detailed report-level judgments across the six QUIPS domains are presented in Table S12 of the Supplementary Materials.

### 2.8. Data Synthesis

The synthesis was organized by prognostic marker and by clinically distinct outcome. Report-specific event data, effect estimates, 95% confidence intervals, and statistical methods were retained in the form reported by the original authors, with univariable and adjusted analyses clearly distinguished. Survival analyses were summarized using the reported time-to-event effect estimates whenever available.

A pooled meta-analysis was not undertaken because heterogeneity concerned not only effect magnitude but also the definition of the prognostic exposures and clinical endpoints themselves. TB definitions, counting methods, and thresholds varied substantially, while WPOI analyses used different category groupings and, in some reports, non-equivalent WPOI-5 constructs. Cervical nodal outcomes were also ascertained through distinct pathways, including elective pathological neck assessment, sentinel lymph node biopsy, and postoperative surveillance, and could not be assumed to represent a common endpoint. Additional heterogeneity in TNM edition, follow-up, effect measures, covariate adjustment, and partially overlapping cohorts further limited comparability. Pooling across these clinically and methodologically non-equivalent data was therefore judged likely to produce a summary estimate of uncertain clinical meaning. Findings were consequently synthesized narratively by prognostic marker, outcome, and cervical-management pathway, with direct quantitative comparisons restricted to clinically and methodologically comparable data.

A restricted subgroup meta-analysis was also considered for studies using commonly applied TB thresholds, particularly the <5- versus ≥5-bud classification. However, a shared numerical threshold did not ensure sufficient methodological or clinical equivalence: studies still differed in staining and counting methods, field definition, cervical-management pathway, outcome ascertainment, effect measure, and covariate adjustment. Moreover, once studies were restricted to a single clinically comparable outcome, the number of sufficiently comparable and independent cohorts was too limited to support a meaningful pooled estimate.

## 3. Results

### 3.1. Study Selection

**Figure 1:**
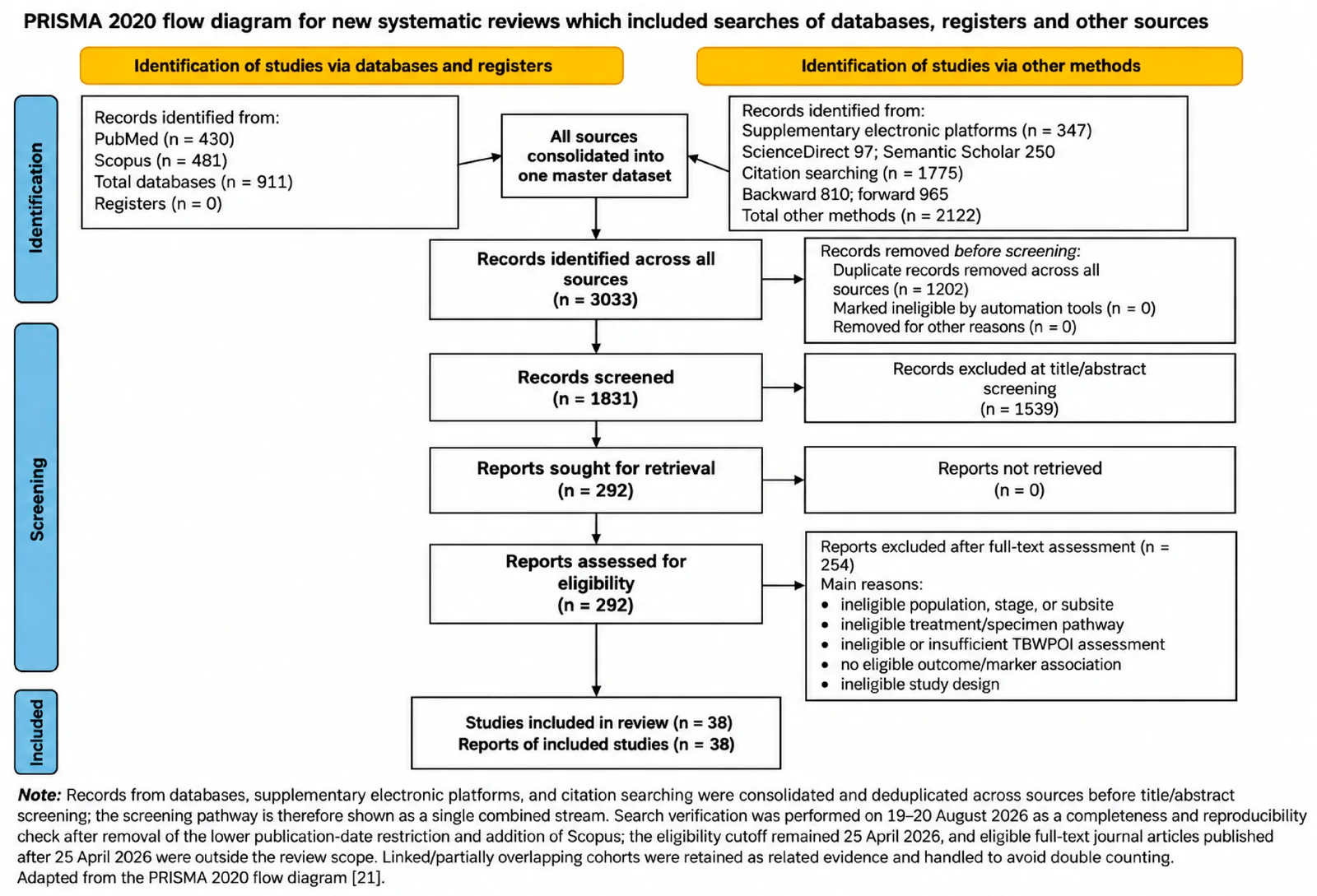
PRISMA 2020 flow diagram of study selection [21].

The search and verification process identified 3033 records: 911 from bibliographic databases (PubMed, *n* = 430; Scopus, *n* = 481) and 2122 from supplementary searches (ScienceDirect, *n* = 97; Semantic Scholar, *n* = 250; backward citation searching, *n* = 810; forward citation searching, *n* = 965). After removal of 1202 duplicate records, 1831 records underwent title and abstract screening, of which 1539 were excluded. Full texts were sought for 292 reports; all were retrieved and assessed for eligibility. Following full-text assessment, 254 reports were excluded and 38 reports were included in the systematic review (Figure 1). Linked or partially overlapping reports were retained only when they contributed non-redundant eligible outcomes or analyses; overlap was handled explicitly in the synthesis to avoid double counting.

### 3.2. Characteristics of the Included Evidence

Thirty-eight reports met the eligibility criteria [32,33,34,35,36,37,38,39,40,41,42,43,44,45,46,47,48,49,50,51,52,53,54,55,56,57,58,59,60,61,62,63,64,65,66,67,68,69]. Because several publications originated from linked or partially overlapping patient cohorts, the evidence base was summarized primarily at the report level, and an unqualified aggregate patient count was not calculated across all reports. The included evidence consisted predominantly of observational cohort studies, most with retrospective designs, with prospective observational cohorts represented less frequently. Study populations focused mainly on the oral tongue or on broader oral-cavity cohorts; other represented subsites included the buccal mucosa, floor of mouth, gingival or alveolar sites, retromolar trigone, palate, and lip. The reports assessed TB, WPOI, or both and used clinical staging frameworks reflecting the period in which the original cohorts were assembled. Detailed report-level characteristics, including study design, sample size, tumor subsite, clinical stage, AJCC/UICC TNM edition, cervical management, and the prognostic marker assessed, are provided in the Supplementary Materials; linked or partially overlapping reports are identified separately to prevent double counting.

### 3.3. Histopathological Assessment of TB and WPOI

Histopathological assessment methods varied substantially across the included reports. Most TB studies defined a bud as an isolated tumor cell or a small cluster of fewer than five tumor cells at the invasive front, but counting methods and thresholds were not uniform. Conventional hotspot-based assessment generally involved low-power scanning followed by counting at ×20, whereas some reports used ×40 high-power fields, cytokeratin immunohistochemistry, or digital/semi-automated image analysis. A dichotomous five-bud threshold (<5 vs. ≥5) was common, but alternative cut-offs included ≥3, ≥4, and ≥6 buds, and several studies used three-tier schemes (0–4, 5–9, and ≥10 buds, or 0, 1–4, and ≥5 buds). WPOI was most often assessed using the Brandwein–Gensler five-tier framework, but analytical groupings varied, including WPOI-1–3 versus WPOI-4–5, WPOI-1–4 versus WPOI-5, and individual WPOI categories. Importantly, the WPOI-5 definition used in the linked Cork reports [55,65] incorporated extratumoral perineural invasion (PNI) and lymphovascular invasion (LVI) in addition to satellite tumor nodules separated by at least 1 mm and was therefore not directly interchangeable with the conventional satellite-based WPOI-5 definition. Reporting of staining, field area, observers, blinding, and consensus procedures remained inconsistent. Detailed report-level definitions, staining methods, scoring procedures, cut-offs, and observer information are provided in Table S7 of the Supplementary Materials.

### 3.4. Clinical Staging and Treatment Characteristics

Clinical staging definitions reflected the TNM classification in use during the respective study periods, and the TNM edition was not consistently reported across the included reports. All eligible cohorts were treated surgically with curative intent, although the extent and description of primary-tumor surgery varied according to tumor subsite and institutional practice. Cervical management was heterogeneous. Where sufficiently described at report level, END was used systematically in six reports, sentinel-node-based staging in two reports, and observation or mixed END/observation strategies in 18 reports; cervical management was insufficiently detailed or variably reported in the remaining 12 reports. This heterogeneity was clinically relevant because pathological nodal involvement identified after END, sentinel-node positivity, and delayed cervical metastasis during surveillance represent distinct endpoints and were therefore synthesized separately. Detailed staging, primary-treatment, and cervical-management characteristics are provided in Table S5 of the Supplementary Materials.

Follow-up, postoperative surveillance, and adjuvant treatment were incompletely and heterogeneously reported across the included reports (Table S11). Directly reported follow-up summaries included a mean of 72.4 ± 11.5 months in Arora et al. [38], a median of 3.0 years (range 0.79–7.6) in the Pedersen cohort [40], 53 months in Sakata et al. [42], 11 months (range 1–42) in Yamanouchi et al. [44], 43 months (range 6–121) in Ebihara et al. [45], 64 months in Heerema et al. [37], 73.2 months (range 7–207) in Kakuguchi et al. [58], and 60 months (mean 72 months) in Hintze et al. [65]. Directly verified longer-term follow-up also included a median of 68 months (range 8–201) in Hori et al. (2020) [50], while Alonso et al. [67] required a minimum postoperative follow-up of 3 years. Several cohorts used observation of the clinically node-negative neck with salvage neck dissection for delayed nodal recurrence, whereas others used systematic END, sentinel-node staging, or mixed strategies. Kakuguchi et al. [58] provided the most detailed surveillance protocol, with progressively lengthened clinical intervals and scheduled cross-sectional imaging during the first two postoperative years. Adjuvant treatment was sparsely reported and was retained as a contextual factor rather than treated as interchangeable outcome data.

### 3.5. Cervical Nodal Outcomes

Because cervical nodal disease was ascertained through different clinical pathways, pathological or occult nodal involvement identified at initial or elective neck assessment, sentinel lymph node biopsy (SLNB) positivity, and delayed cervical metastasis or regional nodal recurrence during postoperative surveillance were synthesized separately. The principal findings are summarized in Table 1; detailed report-level data are provided in Supplementary Table S8.

Reports with mixed or incompletely described cervical ascertainment are retained in Table S8 but were not treated as interchangeable evidence across these endpoint categories.

#### 3.5.1. Pathological or Occult Nodal Disease After Initial/Elective Neck Assessment

Among cohorts with pathological nodal assessment, higher-grade TB showed a consistent adjusted association with cervical nodal involvement. In the prospective cohort of Singh et al. [63], high TB (≥5 buds) and WPOI-4/5 were both independently associated with pathological lymph-node metastasis (OR 30.8, 95% CI 12.365–76.731; *p* < 0.001 and OR 4.5, 95% CI 1.853–11.305; *p* = 0.001, respectively).

Unadjusted and stratified analyses supported an association in several additional cohorts, but the magnitude and independence of WPOI effects were not uniform. Dey et al. [61] reported occult nodal metastasis in 26/102 patients, with node-positive proportions of 21.7% for WPOI-1–3, 23.0% for WPOI-4, and 62.5% for WPOI-5 (*p* = 0.043). In Lakhera et al. [54], nodal positivity occurred in 20.9% of WPOI-1–3 versus 45.6% of WPOI-4/5 tumors (*p* = 0.019 for aggressive WPOI and occult nodal metastasis). Beute et al. [56] found a marked association between WPOI-5 and occult metastasis in pT1 tumors (50.0% vs. 5.7% for WPOI-3/4; *p* < 0.0001), but not in pT2 tumors, and depth of invasion (DOI) ≥4 mm was the dominant predictor. Conversely, Larson et al. [48] found no higher occult-metastasis rate attributable to WPOI-5 within either DOI stratum. In the END subgroup of Hintze et al. [65], the expanded WPOI-5 definition was not associated with occult pN+ disease (*p* = 0.38), whereas LVI remained independently associated (OR 3.6, 95% CI 1.2–10.9; *p* = 0.026). These findings indicate that WPOI associations with occult nodal disease were definition- and cohort-dependent and did not consistently persist after adjustment. Alonso et al. [67] provided a distinct pathological endpoint because all 61 cT1–2 cN0 oral-tongue SCC patients underwent elective neck dissection: 19/27 high-TBI tumors were pN+ (70.4%) compared with 2/34 low-TBI tumors (5.9%; *p* < 0.001). In binary logistic regression, TBI was the only independent predictor (95% CI 1.19–116.68; *p* = 0.035), although the point OR was not printed in the primary report.

#### 3.5.2. Sentinel Lymph Node Biopsy Positivity

Salzano et al. [53] was the only included report in which the eligible nodal endpoint was defined specifically by SLNB positivity. Among 106 cN0 T1–2 oral tongue SCC patients, 35 (33.0%) had a positive SLNB. The proportion positive within each WPOI category was 22.2% for WPOI-I (2/9), 17.2% for WPOI-II (5/29), 21.9% for WPOI-III (7/32), 56.5% for WPOI-IV (13/23), and 61.5% for WPOI-V (8/13). Increasing WPOI class correlated positively with SLNB positivity (Spearman correlation coefficient = 0.342; *p* < 0.001). Pedersen et al. [40] also used sentinel-node biopsy in all source-cohort patients, but its N+ definition combined nodal metastasis detected during primary treatment with isolated subsequent nodal recurrence; that study was therefore retained under mixed nodal ascertainment rather than treated as a pure SLNB-positivity endpoint.

#### 3.5.3. Delayed Cervical Metastasis and Regional Nodal Recurrence

Several surveillance-based cohorts showed that TB was associated with subsequent cervical nodal failure. In Hori et al. (2017) [39], patients treated by primary surgery without END had neck recurrence in 8/13 (62%) high-TB tumors compared with 1/35 (3%) low-TB tumors; high TB remained an independent time-to-event predictor (Cox risk ratio 24.07, 95% CI 2.27–254.89; *p* < 0.01). In the larger multi-institutional series of Yamakawa et al. [46], high TB (≥5 buds) independently predicted delayed neck metastasis (HR 2.22, 95% CI 1.15–4.30; *p* = 0.0179), whereas intermediate TB did not. Sakata et al. [42] studied 97 cT2N0 tongue SCC patients treated by transoral partial glossectomy without END; occult neck metastasis occurred in 16/97 patients, and TB ≥ 4 buds was independently associated with this endpoint (adjusted OR 9.014, 95% CI 1.563–89.075; *p* = 0.012). A linked 62-patient Kanagawa report by Hori et al. (2020) [50] found both WPOI (HR 3.67, 95% CI 1.12–14.06; *p* < 0.05) and the BD model (HR 5.46, 95% CI 1.08–27.52; *p* < 0.05) independently predictive of regional control. The BD-positive definition combined tumor depth ≥ 3 mm with TB ≥ 5. Hori et al. (2021) [51], using the same or substantially overlapping Kanagawa population, found intermediate/high TB independently associated with poorer regional control (HR 17.91, 95% CI 2.28–140.73; *p* = 0.006). These linked Hori reports were therefore not interpreted as independent replications. Yamazaki et al. [60] evaluated 69 patients with Stage I/II tongue SCC treated by partial glossectomy and postoperative observation. TB ≥ 6 and WPOI-5 showed borderline univariable associations with secondary lymph-node metastasis (HR 2.76, 95% CI 0.94–8.09; *p* = 0.06 and HR 2.85, 95% CI 0.91–8.95; *p* = 0.07, respectively), but neither marker was retained in the multivariable model.

WPOI and TB were not uniformly independent in surveillance cohorts. In Kakuguchi et al. [58], cervical lymph-node metastasis occurred in 23/89 patients; TB ≥ 5 and WPOI-4/5 were associated with shorter nodal-metastasis-free survival by log-rank testing (*p* = 0.009 and *p* = 0.003, respectively), but neither was retained as an independent predictor in the final Cox model, in which Yamamoto–Kohama (YK)-4c invasion and pathological DOI > 5 mm were strongest. The *p* = 0.003 result for WPOI therefore represents a time-to-event log-rank comparison rather than a crude comparison of proportions. Yamauchi et al. [59] reported significantly more regional nodal recurrence with WPOI-4/5 than WPOI-1–3 after primary resection without END; because a numerical effect estimate could not be verified directly in the primary report, the association was retained qualitatively. In Obayashi et al. [62], no late cervical metastasis occurred among tumors with <3 buds (0/31) compared with 15/30 tumors with ≥3 buds (*p* < 0.0001); TB remained significant in multivariable analysis, although the numerical coefficient was unstable because of the zero-event cell.

Hintze et al. [65] examined regional recurrence in a mixed pN0/pNx cohort rather than an exclusively untreated neck. In the combined 170-patient pN0/pNx analysis, expanded WPOI-5 independently predicted isolated regional recurrence (OR 5.21, 95% CI 1.24–21.98) and any regional recurrence (OR 8.53, 95% CI 1.76–41.41). This evidence was retained but interpreted separately because the WPOI-5 definition included extratumoral PNI/LVI and because the cohort combined patients with and without prior neck dissection. Kikuchi et al. [66] reported late nodal metastasis in 17/66 patients (25.8%). Using a ROC-derived cut-off of ≥6 buds, TB remained an independent predictor (adjusted OR 3.78, 95% CI 1.19–12.10; *p* = 0.025); the median time to late nodal metastasis was 10 months (range 2–26).

#### 3.5.4. Nodal Outcomes with Mixed Cervical Ascertainment

Several reports combined pathological neck assessment with postoperative surveillance or did not provide a single uniform cervical pathway. Arora et al. [38] included 336 cT1/2N0M0 OSCC patients: 181 underwent prophylactic selective neck dissection, with 52 pN+ cases, whereas 155 were initially observed and 40 subsequently underwent delayed modified radical neck dissection for clinically evident nodal relapse; high TB remained independently associated with nodal metastasis (adjusted OR 1.28, 95% CI 1.09–2.61; *p* = 0.039). Ebihara et al. [45] used wait-and-watch in 42/64 patients and END in 22/64; 21/64 ultimately had pathological nodal metastasis, and TBG3 (≥10 buds) independently predicted NLM (OR 9.55, 95% CI 1.80–50.8; *p* = 0.008). Similarly, Sakai et al. [52] evaluated 70 cT1/2N0 oral-tongue SCCs managed with END in 16 patients and without END in 54. TB was associated with nodal metastasis on univariable analysis (*p* = 0.008) but was not retained in the multivariable model, in which pathological DOI and tumor–stroma ratio remained significant. Goyal et al. [68] reported pathological nodal status in 50 T1/T2 cN0 OSCC patients. TB ≥ 5 and WPOI-5 were each associated with nodal positivity (*p* = 0.04), although no adjusted marker-specific model was reported; because the exact cervical surgical procedure was not explicitly described, the study was retained in this ascertainment category. Calabrese et al. [69] included 1109 cT1–2 cN0 oral cavity SCC patients: 682 underwent END and 427 were managed without END using clinical and ultrasound surveillance. Among patients undergoing END, 193/682 (28.3%) were pN+. In the univariable model, source-defined high-risk WPOI was associated with occult nodal disease (OR 4.85, 95% CI 2.95–8.07; *p* < 0.001), but WPOI was available in only 301/1109 patients and was therefore not entered into the final multivariable model. Zheng et al. [64] used END in 57/71 patients and a wait-and-watch strategy in 14/71; overall, occult cervical metastasis was identified in 20/70 evaluable patients (28.6%), with increased adjusted odds for TBG2 versus TBG1 (OR 6.08, 95% CI 1.50–24.62; *p* = 0.011). Xie et al. [36] reported cervical nodal status for 168/195 cT1/2N0M0 tongue SCCs; primary tumor resection and/or END were used, but the exact END-versus-surveillance distribution was not reported, so this nodal evidence was retained in the mixed/insufficiently described pathway stratum.

### 3.6. Recurrence Outcomes

Recurrence outcomes were heterogeneous across the included reports. Because delayed or isolated regional nodal failures have already been synthesized under cervical nodal outcomes, the present section focuses on local recurrence, composite locoregional recurrence (LRR), and locoregional recurrence-free survival (LRRFS). DFS/RFS, DSS/CSS, and OS outcomes are summarized separately below. A recurrence-defined DFS analysis reported by Dourado et al. is retained in Supplementary Table S9 for source-level completeness but is synthesized with the survival outcomes in Section 3.7. The principal findings related to recurrence outcomes are summarized in Table 2; detailed report-level data are provided in Supplementary Table S9.

The clearest adjusted evidence for conventional WPOI was provided by Li et al. [32] in 292 analyzed patients with Stage I/II oral cavity SCC. Fifty-six T1/T2N0 patients developed LRR. After adjustment for potential confounders, WPOI predicted LRR using either a WPOI-4 cut-point (HR 3.63, 95% CI 1.56–8.47; *p* = 0.0029) or a WPOI-5 cut-point (HR 2.55, 95% CI 1.48–4.41; *p* = 0.0008); the reported probability of LRR in WPOI-5 tumors was 42%. Heerema et al. [37] likewise found that the predominant pattern of invasion and the summed predominant-plus-worst pattern were independent prognosticators of LRRFS (HR 2.1, *p* = 0.033 and HR 2.2, *p* = 0.024, respectively), although interobserver reproducibility of pattern-of-invasion scoring was only moderate.

Results from the linked Almangush cohorts require interpretation as related rather than independent evidence. In the 311-patient multicenter cohort [34], the high-risk budding–depth (BD) score, combining TB ≥ 5 with DOI ≥ 4 mm, independently predicted LRR (*p* = 0.033); because a recurrence-specific numerical effect estimate could not be verified directly in the primary report, no secondary numerical estimate was substituted. In the expanded 479-patient international cohort [35], DOI ≥ 4 mm remained associated with LRR (adjusted HR 1.67, 95% CI 1.07–2.60), whereas the adjusted estimate for high-risk WPOI was HR 1.46 (95% CI 0.95–2.25), with the confidence interval including unity. Thus, the latter report does not demonstrate a statistically independent WPOI effect on LRR after adjustment.

TB-specific recurrence evidence was also reported in several independent cohorts. In Xie et al. [36], Table 1 of the primary report showed local relapse in 3 low-intensity and 19 high-intensity TB tumors (22/195 overall), with a significant association between TB intensity and local relapse (*p* = 0.001); no recurrence-specific adjusted effect estimate was reported. In Zheng et al. [64], 10/71 patients developed locoregional recurrence. In the primary multivariable Table 3, TBG2 was not associated with LRRFS (HR 1.01, 95% CI 0.14–7.54; *p* = 0.992), whereas TBG3 was associated with poorer LRRFS (HR 12.38, 95% CI 1.58–96.75; *p* = 0.016); the overall trend test was not statistically significant (*p* = 0.062). These table-level estimates were retained in preference to discrepant summary values in the abstract and narrative Results.

Other reports illustrate why WPOI-related recurrence effects should not be generalized across scoring systems. In Sinha et al. [41], a higher WPOI score was associated with recurrence on univariable analysis (*p* = 0.032), whereas WPOI-5/multifocality itself was not (*p* = 0.524); the adjusted OR of 12.4 (*p* = 0.001) applied to the composite Brandwein–Gensler high-risk category rather than WPOI alone. In Larson et al. [48], WPOI-5 alone was not associated with higher 2-year LRR within either DOI stratum; only the presence of at least two adverse features among WPOI-5, PNI, and LVI independently increased LRR (OR 5.7; *p* = 0.01).

In the Cork cohort reported by Mohamed et al. [55], the expanded WPOI-5 definition was strongly associated with LRR on univariable analysis (OR 6.77, 95% CI 2.89–15.84; *p* < 0.0001). However, when the overlapping WPOI-5 components were modeled, the independent LRR predictors were extratumoral LVI (OR 13.49, 95% CI 3.13–56.99; *p* = 0.0004), extratumoral PNI (OR 4.65, 95% CI 1.17–18.54; *p* = 0.03), and any PNI (OR 2.92, 95% CI 1.23–6.93; *p* = 0.02), rather than the composite WPOI-5 variable. Fonseca et al. [57] also found an overall association between the WPOI category and LRR on univariable log-rank analysis (*p* < 0.00001). Among 207 T1N0M0/T2N0M0 patients, 113 had WPOI-1–3 (45 recurrences), 91 had WPOI-4 (67 recurrences), and only three had WPOI-5 (one recurrence). Because the WPOI-5 subgroup was very small and no category-specific adjusted effect estimate was reported, these data do not establish a WPOI-5-specific recurrence effect.

### 3.7. Survival Outcomes

Survival endpoints were retained as distinct outcomes according to the definitions used by the original reports. Eligible endpoints comprised disease-free survival (DFS), recurrence-free survival (RFS), disease-specific survival (DSS), cancer-specific survival (CSS), and overall survival (OS). DSS and CSS were interpreted within the same disease-specific mortality domain while preserving the terminology used by each study, whereas DFS and RFS were kept separate when definitions differed. Progression-free survival (PFS) was not an eligible endpoint. When time-to-event effect estimates were available, adjusted estimates were prioritized over crude proportions. The principal findings are summarized in Table 3; detailed report-level data are provided in Supplementary Table S10.

For disease-specific mortality, Li et al. [32] provided strong adjusted evidence for conventional WPOI in low-stage oral cavity SCC. Among T1/T2N0 patients, disease-specific mortality occurred in 18 patients; WPOI-5 independently predicted DSS (HR 6.34, 95% CI 2.50–16.09; *p* = 0.0001). In the linked Finnish early-oral tongue SCC cohort reported by Almangush et al. [33], both high TB (≥5 buds) and high-risk WPOI were independently associated with mortality from oral tongue SCC after adjustment: TB HR 2.04 (95% CI 1.17–3.55; *p* = 0.01) and WPOI HR 4.47 (95% CI 1.59–12.51; *p* = 0.004). The subsequent linked BD-model report [34] also found the high-risk combined score to predict death due to oral tongue SCC (*p* < 0.001), whereas the expanded 479-patient international analysis [35] identified high-risk WPOI as an independent predictor of disease-related mortality (HR 2.34, 95% CI 1.26–4.32). These reports arise from partially overlapping cohorts and therefore represent related analyses rather than independent replications.

TB was independently associated with survival in several other early-stage cohorts. Pedersen et al. [40] used semi-automated digital tumor bud count (DTBC) in 222 early-stage OSCCs and found a dose–response relationship with OS: compared with the lower tertile, the upper tertile had an unadjusted HR of 4.0 (95% CI 1.9–8.4), while DTBC remained independently associated with OS when modeled per tertile increase (HR 1.6, 95% CI 1.1–2.2; *p* = 0.01). Xie et al. [36] likewise found that high-intensity TB independently predicted poorer OS in cT1/2N0M0 tongue SCC; survival follow-up was available for 106/195 patients, and the adjusted Cox estimate for high versus low TB was HR 5.582 (95% CI 1.227–25.381; *p* = 0.026).

DFS showed a similar but not uniform pattern. In Shimizu et al. [43], five-year DFS was 65.0%, 37.5%, and 23.1% for low-, intermediate-, and high-intensity TB, respectively (*p* < 0.01), and 58.4% versus 33.3% for WPOI-1–4 versus WPOI-5 (*p* = 0.03). In the multivariable Cox model, TB intensity remained independently associated with DFS (HR 2.18, 95% CI 1.49–3.20; *p* < 0.01), whereas WPOI did not remain in the final model. Yamanouchi et al. [44] similarly reported longer DFS for low- versus high-grade TB (*p* = 0.047). Parekh et al. [49] found univariable associations of WPOI-4/5 and high TB with poorer DFS, but only WPOI-4 remained independently prognostic (HR 5.019, 95% CI 1.212–20.789; *p* = 0.026). In the early-stage subgroup of Dourado et al. [47] (*n* = 103), TB was independently associated with worse cancer-specific survival (HR 3.03, 95% CI 1.30–7.07; *p* = 0.01), whereas its association with DFS was not statistically significant (HR 1.98, 95% CI 0.92–4.27; *p* = 0.07). In Hori et al. (2020) [50], WPOI independently predicted DFS (HR 3.84, 95% CI 1.30–11.34; *p* < 0.05), whereas the BD model was not retained for DFS.

The linked Cork report by Mohamed et al. [55] used the expanded WPOI-5 definition incorporating satellite nodules and extratumoral PNI/LVI. WPOI-5 and each of its three components were significantly associated with DSS and OS on univariable analysis. DSS could not be modeled multivariably because only 15 disease-specific deaths occurred. For OS, when WPOI-5 was analyzed without its overlapping components, it remained strongly associated with mortality (OR 7.57, 95% CI 3.67–15.60); in the model containing the individual components instead, satellite nodules (OR 6.61, 95% CI 2.83–15.44; *p* < 0.0001) and extratumoral LVI (OR 9.97, 95% CI 2.19–45.35; *p* = 0.003) were independently associated with OS. These findings should therefore be interpreted in the context of the expanded WPOI-5 construct and substantial overlap among its components.

Alonso et al. [67] did not find a significant TBI association with time to locoregional recurrence/DFS (93.9 ± 7.0 vs. 74.8 ± 10.2 months; *p* = 0.076) or OS (93.2 ± 7.6 vs. 83.9 ± 9.3 months; *p* = 0.219). Overall, survival findings were directionally consistent with an adverse prognostic role for high TB and non-cohesive/WPOI-5 invasion in several cohorts, but the independent marker retained after adjustment varied across studies. Differences in endpoint definitions, marker cut-offs, WPOI constructs, adjustment sets, and linked cohorts precluded pooling of DFS/RFS, DSS/CSS, or OS in the present review.

### 3.8. Risk of Bias

Risk of bias varied across reports and QUIPS domains (Table S12). After final eligibility verification and primary-source reappraisal, the report-level matrix contained 38 reports. High-risk classifications were most frequent for prognostic factor measurement (10/38) and study confounding (8/38), followed by outcome measurement (4/38), study participation (4/38), statistical analysis and reporting (3/38), and study attrition (2/38). No overall numerical QUIPS score was calculated.

## 4. Discussion

This systematic review evaluated the prognostic relevance of tumor budding and worst pattern of invasion in surgically treated patients with cT1–2N0M0 squamous cell carcinoma of the lip and oral cavity. The expanded evidence base comprised 38 eligible reports. Several publications arose from linked or partially overlapping cohorts and were therefore interpreted as related analyses rather than independent replications. Across this expanded evidence base, higher-risk WPOI and high TB were associated with clinically relevant cervical nodal, recurrence, and survival outcomes. However, the independent effect retained after adjustment varied by endpoint, marker definition, and statistical model, emphasizing the heterogeneity of the available prognostic evidence.

One of the motivations for the present review arose from our previous institutional cohort, which showed persistent heterogeneity in outcomes despite evaluation of conventional clinicopathological prognostic factors [70]. TB and WPOI had not been systematically evaluated in that historical cohort, highlighting the potential value of additional invasive-front markers for explaining residual prognostic heterogeneity.

WPOI describes the architecture of the invasive tumor front and may reflect the ability of malignant cells to detach from the primary tumor and infiltrate the surrounding tissues. WPOI-4 is characterized by small tumor islands, strands, or individual cells, whereas WPOI-5 is defined by satellite tumor deposits separated from the primary tumor or the nearest satellite by at least 1 mm. The association of these patterns with adverse outcomes is consistent with systematic reviews and meta-analyses reporting relationships between non-cohesive invasion, nodal metastasis, locoregional recurrence, and poorer survival [4,5,10,13].

However, WPOI assessment was not uniform across the included studies. Some authors analyzed all five categories separately, whereas others grouped WPOI-1–3 as cohesive and WPOI-4–5 as non-cohesive or invasive patterns. Moreover, Hintze et al. used a study-specific definition of WPOI-5 that also incorporated extratumoral perineural or lymphovascular invasion [65]. TB and WPOI-4 both reflect aspects of non-cohesive invasion but differ in their counting units, thresholds, and assessment methods and should not be considered interchangeable. These differences limit direct comparisons and may partly explain the variability in reported effect estimates [6,7,8].

Evidence for TB was substantially broader in the expanded evidence base. Across eligible early-stage cohorts, high TB was associated with pathological or occult nodal disease, delayed cervical metastasis, local or locoregional recurrence, and adverse survival outcomes. However, independence was not uniform: some multivariable models retained TB as a prognostic factor, whereas others showed attenuation after adjustment for WPOI, DOI, or other clinicopathological covariates. This pattern is consistent with previous systematic reviews and meta-analyses linking high-grade budding with nodal metastasis and adverse survival in OSCC [4,5,6,7,9,11,13,14,15,16,17], but it also indicates that TB should not be interpreted as a stand-alone predictor. Variation in bud definitions, thresholds, field size, manual versus digital assessment, and adjustment sets further limits direct cross-study comparison. Several apparently large effect estimates were accompanied by very wide confidence intervals, reflecting small samples and/or sparse outcome events. These estimates should therefore be interpreted primarily as evidence regarding the possible direction of an association rather than as stable or precise estimates of its magnitude.

Importantly, this heterogeneity affects more than the feasibility of statistical pooling. A designation such as “high TB” or “high-risk WPOI” did not necessarily represent an equivalent histopathological exposure across studies. Differences in bud definition, counting field, staining method, analytical threshold, WPOI grouping, and, in some reports, the components included within WPOI-5 mean that effect estimates may refer to biologically and methodologically non-equivalent constructs. The direction and magnitude of associations should therefore be interpreted within the specific marker definition used in each study rather than as estimates of a single uniform prognostic exposure.

TB and WPOI are correlated with other adverse pathological characteristics, including DOI, PNI, LVI, differentiation, and related measures of invasive phenotype. Consequently, a univariable association does not establish independent prognostic value, and even an adjusted association depends on adequate model specification and control of clinically relevant confounders. In the present evidence base, the marker retained after adjustment varied among studies, while QUIPS identified study confounding as one of the most frequent high-risk domains. Claims of marker independence should therefore be restricted to appropriately adjusted analyses and interpreted cautiously.

The strength of the observed prognostic associations must also be interpreted in light of risk of bias. QUIPS concerns were most frequent for prognostic-factor measurement and study confounding, precisely the domains most relevant to the comparability and claimed independence of TB/WPOI effects. Accordingly, concordant associations across reports should not be interpreted as uniformly high-confidence evidence when marker measurement was non-standardized or control of relevant confounding was incomplete.

The clinical interpretation of these markers should also account for differences in cervical management. Outcomes were determined through elective neck dissection, sentinel lymph node biopsy, or postoperative surveillance, depending on the study. These approaches identify nodal disease at different time points and are not directly interchangeable. Postoperative follow-up and adjuvant management represented an additional source of clinical heterogeneity, because surveillance intensity and salvage or adjuvant treatment were inconsistently reported and may have influenced both the timing of event detection and subsequent recurrence or survival outcomes independently of TB/WPOI. Furthermore, because eligibility required assessment on resection specimens, the present findings primarily inform postoperative risk stratification and do not establish the preoperative utility of TB or WPOI for selecting the initial cervical treatment. Consequently, these markers should not currently be used as isolated determinants of elective neck treatment, adjuvant therapy, or surveillance intensity. Instead, they may provide complementary information when interpreted together with depth of invasion, perineural and lymphovascular invasion, tumor differentiation, resection margin status, and pathological nodal stage.

Both markers can generally be evaluated on hematoxylin-and-eosin-stained sections, supporting their potential clinical applicability. Nevertheless, observer methodology was inconsistently reported, and interobserver agreement was generally not evaluated. TB assessment may be particularly difficult in areas with marked inflammation or stromal reaction, where isolated tumor cells can be difficult to distinguish from non-neoplastic elements; cytokeratin immunohistochemistry may assist in selected difficult cases, although its routine role remains uncertain [6,18,19,20]. Standardized observer training, clearly defined counting methods, and reproducibility testing are therefore required before widespread implementation.

The strengths of this review include its focused clinical population, restriction to surgically treated clinically node-negative early-stage disease, the absence of a lower publication-date restriction in the search strategy, detailed assessment of cervical management and pathological methodology, explicit handling of overlapping cohorts, and structured risk-of-bias evaluation using QUIPS. Nevertheless, several limitations remain.

The review was not prospectively registered in PROSPERO, and no separate prespecified protocol was prepared. Although the review methods and subsequent search-verification procedures are reported transparently, the absence of prospective registration limits independent verification that all methodological and analytical decisions were specified a priori and should therefore be considered a limitation of the review process.

Embase was not searched, which may have reduced search sensitivity for studies indexed uniquely or more readily retrievable in that database. This limitation was partly mitigated, but cannot be considered eliminated, by the use of PubMed and Scopus as bibliographic databases, supplementary searches in ScienceDirect and Semantic Scholar, and extensive backward and forward citation searching. Although 38 reports met the eligibility criteria, the effective amount of independent evidence was smaller because several publications represented serial, extended, or partially overlapping patient cohorts. Together with the predominance of retrospective designs, this reduces the effective independence of the evidence base and limits the extent to which apparently concordant findings can be interpreted as independent replication. Marker definitions, thresholds, clinical outcomes, follow-up protocols, and statistical methods were heterogeneous, preventing a single quantitative synthesis. Reporting of observer procedures, adjuvant therapy, missing data, and relevant confounders was incomplete.

Oral tongue SCC was disproportionately represented, whereas other oral-cavity and lip subsites contributed substantially fewer data. Because anatomical subsites differ in patterns and propensity of lymphatic dissemination and may also differ in local invasive behavior, the present findings cannot be assumed to have identical prognostic implications across all lip and oral-cavity subsites. Subsite-specific validation is therefore required.

The restriction to English-language full-text articles may have excluded some relevant evidence. The literature eligibility cutoff was 25 April 2026; therefore, reports published online or in print as eligible full-text journal articles subsequently were outside the scope of the present review. The QUIPS assessment identified the most frequent high-risk judgments in prognostic factor measurement and study confounding, with less frequent high-risk judgments in outcome measurement, study participation, study attrition, and statistical analysis/reporting.

Future studies should use prospective multicenter designs, standardized definitions and thresholds, predefined clinical outcomes, and uniform reporting of cervical staging and treatment. WPOI-4 and WPOI-5 should preferably be reported separately, and TB assessment should include a clearly specified field size, counting method, and cut-off value. Survival endpoints should also be defined consistently, with DFS, RFS, DSS/CSS, and OS reported separately and accompanied by adjusted time-to-event estimates whenever appropriate. Studies should evaluate interobserver reproducibility and determine whether these markers provide prognostic information beyond DOI, PNI, LVI, margins, and conventional TNM staging. At present, both WPOI and TB appear potentially useful as complementary postoperative prognostic features, but neither should be used in isolation.

## 5. Conclusions

This systematic review identifies recurrent associations between adverse invasive-front morphology and clinically relevant outcomes in patients with early-stage cT1–2N0M0 squamous cell carcinoma of the lip and oral cavity. Higher TB and higher-risk WPOI were associated with cervical nodal involvement, delayed nodal failure, recurrence, and adverse survival in multiple reports; however, the consistency, magnitude, and independence of these associations varied across studies.

Confidence in these prognostic associations is limited by heterogeneous and sometimes non-equivalent marker definitions; predominantly retrospective study designs; partially overlapping cohorts; incomplete or variable adjustment for DOI, PNI, LVI and other pathological factors; and risk-of-bias concerns, particularly in prognostic-factor measurement and study confounding. Accordingly, the available evidence suggests potential complementary prognostic information from TB and WPOI but does not establish either marker as a uniform or independent prognostic determinant across early-stage lip and oral cavity SCC.

TB and WPOI should therefore be interpreted as complementary histopathological features rather than isolated determinants of treatment. Their interpretation should remain integrated with depth of invasion, perineural and lymphovascular invasion, resection margin status, tumor differentiation, nodal status, and other relevant clinicopathological factors.

Prospective, multicenter studies using standardized definitions, scoring systems, observer training, and predefined nodal, recurrence, and survival outcomes are needed to determine how these markers can be incorporated consistently into routine pathology reporting and therapeutic decision-making. Validation of combined prognostic models may ultimately improve postoperative risk stratification. Any role in selecting patients for initial elective neck treatment would require separate validation using preoperative specimens.

## Figures and Tables

**Table 1:** Summary of tumor budding and worst pattern of invasion associations with cervical nodal outcomes.

Clinical Pathway/ Endpoint	Marker/Evidence Base	Summary of Findings
Pathological/occult nodal disease after initial or elective neck assessment	TB/TBI— Singh [63], Alonso [67]	Higher TB/TBI was strongly associated with pathological nodal involvement and remained independently associated in both studies. Singh [63] reported adjusted OR 30.8 (95% CI 12.365–76.731; *p* < 0.001). Alonso [67] identified TBI as the only independent predictor (*p* = 0.035), although the 95% CI was very wide (1.19–116.68; point OR not reported).
	WPOI— Singh [63], Dey [61], Lakhera [54], Beute [56], Larson [48], Hintze [65]	Higher-risk WPOI was associated with occult/pathological nodal disease in several cohorts, but findings were not uniform. WPOI-4/5 remained independently associated in Singh [63]; Dey [61] and Lakhera [54] were unadjusted, Beute [56] was pT/DOI-dependent, Larson [48] was negative, and expanded WPOI-5 was not associated with occult pN+ disease in Hintze [65], where LVI remained independently associated.
Sentinel lymph-node biopsy positivity	WPOI— Salzano [53]	Increasing WPOI class was associated with higher SLNB positivity, particularly for WPOI-IV/V (Spearman coefficient = 0.342; *p* < 0.001), but no adjusted WPOI-specific effect was reported. Salzano [53] was the only pure SLNB-positivity report; Pedersen [40] used a composite N+ endpoint and was retained under mixed ascertainment.
Delayed/late cervical metastasis or regional nodal failure during postoperative surveillance	TB/TBG— Hori 2017 [39], Sakata [42], Yamakawa [46], Hori 2021 [51], Yamazaki [60], Kakuguchi [58], Obayashi [62], Kikuchi [66]	Most surveillance-based reports showed an adverse association between higher TB and subsequent cervical nodal failure. Independent associations were reported by Hori 2017 [39], Sakata [42], Yamakawa [46], Hori 2021 [51], Obayashi [62], and Kikuchi [66]; Yamazaki [60] showed only a borderline univariable association, and Kakuguchi [58] did not retain TB after adjustment. Effect magnitude and precision varied substantially; linked Hori reports are not independent replications.
	WPOI/BD-related evidence — Hori 2020 [50], Yamazaki [60], Kakuguchi [58], Yamauchi [59]	WPOI-related features were associated with nodal failure in some reports, but adjusted independence was inconsistent. Hori 2020 [50] reported independent WPOI and BD-model associations; Yamazaki [60] and Kakuguchi [58] did not retain WPOI after adjustment, while Yamauchi [59] provided qualitative evidence only because the numerical effect estimate could not be directly verified.
Delayed/regional nodal recurrence in a mixed pN0/pNx follow-up cohort	Expanded WPOI-5— Hintze [65]	Expanded WPOI-5 independently predicted isolated regional recurrence (OR 5.21, 95% CI 1.24–21.98) and any regional recurrence (OR 8.53, 95% CI 1.76–41.41). It was interpreted separately because the construct incorporated extratumoral PNI/LVI and the cohort included patients with and without prior neck dissection.
Mixed or incompletely described cervical ascertainment	TB/TBG/WPOI— Xie [36], Arora [38], Pedersen [40], Shimizu [43], Ebihara [45], Sakai [52], Zheng [64], Goyal [68], Calabrese [69]	Several reports showed adverse associations, but cervical ascertainment and statistical adjustment varied markedly. Adjusted associations were retained in some cohorts (Arora [38], Ebihara [45], Zheng [64]), whereas other findings were unadjusted, composite, not retained after adjustment, or incompletely modeled. Pedersen [40] used a composite N+ endpoint; Shimizu [43] contained an internal statistical inconsistency; Calabrese [69] could not include WPOI in the final multivariable model because WPOI data were available in only 301/1109 patients.

Note: Cervical nodal outcomes are grouped according to the clinical pathway by which nodal disease was identified. Pathological nodal positivity after elective neck assessment, SLNB positivity, delayed nodal metastasis during surveillance, and mixed ascertainment were considered clinically distinct endpoints. Detailed report-level data, including individual effect estimates, confidence intervals, and statistical interpretation, are provided in Supplementary Table S8. Abbreviations: BD, budding–depth model; CI, confidence interval; DOI, depth of invasion; LVI, lymphovascular invasion; OR, odds ratio; pN+, pathologically node-positive; PNI, perineural invasion; SLNB, sentinel lymph-node biopsy; TB, tumor budding; TBG, tumor budding grade; TBI, tumor budding index; WPOI, worst pattern of invasion.

**Table 2:** Summary of tumor budding and worst pattern of invasion associations with local and locoregional recurrence outcomes.

Evidence Domain	Evidence Base	Summary of Findings
Conventional WPOI/pattern-of-invasion evidence	Li [32], Heerema [37]	The clearest adjusted recurrence evidence for conventional invasive-front morphology was reported in these cohorts. Li [32] found WPOI independently associated with LRR using both WPOI-4 (HR 3.63, 95% CI 1.56–8.47; *p* = 0.0029) and WPOI-5 (HR 2.55, 95% CI 1.48–4.41; *p* = 0.0008) cut-points. Heerema [37] reported independent associations of predominant POI and summed predominant-plus-worst POI with LRRFS, although interobserver reproducibility was only moderate.
Linked Almangush prognostic models (BD model/high-risk WPOI)	Almangush 2015a [34], Almangush 2015b [35]	The high-risk BD score (TB ≥ 5 plus DOI ≥ 4 mm) independently predicted LRR in the 311-patient cohort (*p* = 0.033), whereas in the expanded 479-patient cohort high-risk WPOI was not statistically conclusive after adjustment (HR 1.46, 95% CI 0.95–2.25); DOI ≥ 4 mm remained independently associated. These reports arise from linked/partially overlapping cohorts and are not independent replications.
TB/TBG-related recurrence evidence	Xie [36], Zheng [64]	TB-related recurrence findings were heterogeneous. Xie [36] reported a significant unadjusted association with local relapse (*p* = 0.001) but no recurrence-specific adjusted estimate. Zheng [64] found TBG3 associated with poorer LRRFS (HR 12.38, 95% CI 1.58–96.75; *p* = 0.016), but the estimate was highly imprecise and the overall trend test was not significant (*p* = 0.062).
WPOI within composite histologic risk constructs	Sinha [41], Larson [48]	Positive adjusted recurrence effects were attributable to composite risk constructs rather than WPOI alone. In Sinha [41], WPOI score was associated with recurrence univariably, but WPOI-5/multifocality was not; the adjusted OR 12.4 applied to the composite Brandwein–Gensler high-risk category. In Larson [48], WPOI-5 alone did not increase 2-year LRR; only the presence of ≥2 adverse features among WPOI-5, PNI, and LVI independently predicted LRR (OR 5.7; *p* = 0.01).
Expanded WPOI-5	Mohamed [55]	Expanded WPOI-5 was strongly associated with LRR on univariable analysis (OR 6.77, 95% CI 2.89–15.84; *p* < 0.0001). However, when its overlapping components were modeled, extratumoral LVI, extratumoral PNI, and any PNI remained independent rather than the composite WPOI-5 variable. This study-specific construct is not directly interchangeable with conventional satellite-based WPOI-5.
WPOI category analysis	Fonseca [57]	Overall WPOI category was associated with LRR on univariable log-rank analysis (*p* < 0.00001), but no adjusted WPOI-specific effect was reported. Only three patients had WPOI-5 and one recurred; therefore, the significant overall category association does not establish a WPOI-5-specific recurrence effect.

Note: This table summarizes local recurrence, composite locoregional recurrence (LRR), and locoregional recurrence-free survival (LRRFS) evidence reported in Section 3.6. Delayed or isolated regional nodal failures are summarized with cervical nodal outcomes and are not duplicated here. Supplementary Table S9 additionally retains the recurrence-defined DFS analysis of Dourado et al. for source-level completeness; DFS is synthesized under survival outcomes in Section 3.7. Detailed report-level event data, effect estimates, confidence intervals, and methodological notes are provided in Supplementary Table S9. Abbreviations: BD, budding–depth model; CI, confidence interval; DFS, disease-free survival; DOI, depth of invasion; HR, hazard ratio; LRR, locoregional recurrence; LRRFS, locoregional recurrence-free survival; LVI, lymphovascular invasion; OR, odds ratio; PNI, perineural invasion; POI, pattern of invasion; TB, tumor budding; TBG, tumor budding grade; WPOI, worst pattern of invasion.

**Table 3:** Summary of tumor budding and worst pattern of invasion associations with survival outcomes.

Survival Endpoint/ Evidence Domain	Marker/Evidence Base	Summary of Findings
Disease-specific/cancer-specific survival (DSS/CSS)	WPOI/POI— Li [32], Almangush 2014 [33], Almangush 2015b [35], Heerema [37], Yamauchi [59]	Several cohorts showed adverse associations between higher-risk WPOI/POI and disease-specific mortality. Independent associations were reported by Li [32] (WPOI-5 HR 6.34, 95% CI 2.50–16.09), Almangush 2014 [33] (WPOI HR 4.47, 95% CI 1.59–12.51), Almangush 2015b [35] (HR 2.34, 95% CI 1.26–4.32), and Heerema [37] (POI-based HRs 2.1–2.3). Yamauchi [59] reported 5-year DSS of 100% for WPOI-1–3 versus 56% for WPOI-4/5 (*p* = 0.0001), but only one patient had WPOI-5 and no adjusted effect estimate was reported. Linked Almangush reports are not independent replications.
	TB/TBG/BD-related evidence— Almangush 2014 [33], Almangush 2015a [34], Sakata [42], Ebihara [45], Dourado [47]	Adjusted adverse TB associations were reported by Almangush 2014 [33] (HR 2.04, 95% CI 1.17–3.55), Ebihara [45] (TBG3 HR 4.41, 95% CI 1.34–14.5), and Dourado [47] (CSS HR 3.03, 95% CI 1.30–7.07). Sakata [42] showed a strong univariable association, but the adjusted TB effect was not significant (HR 3.114, 95% CI 0.520–32.052; *p* = 0.232). The linked Almangush [34] BD model also predicted disease-related death, but it represents a composite TB + DOI construct rather than TB alone.
Disease-free survival (DFS)	TB/TBG/TBI— Shimizu [43], Yamanouchi [44], Dourado [47], Hori 2021 [51], Alonso [67]	TB-related DFS evidence was mixed. Shimizu [43] found TB intensity independently associated with poorer DFS (HR 2.18, 95% CI 1.49–3.20), while Yamanouchi [44] reported an unadjusted association (*p* = 0.047). By contrast, TB was not independently associated with DFS in Dourado [47] (HR 1.98, 95% CI 0.92–4.27; *p* = 0.07) or Hori 2021 [51] (HR 1.95, 95% CI 0.79–4.84; *p* = 0.15), and Alonso [67] found no significant TBI association with time to locoregional recurrence/DFS (*p* = 0.076).
	WPOI/BD-related evidence— Shimizu [43], Parekh [49], Hori 2020 [50]	WPOI-related DFS findings were variable. Shimizu [43] found WPOI-5 associated with poorer DFS on Kaplan–Meier analysis, but WPOI was not retained in the final Cox model. Parekh [49] identified WPOI-4 as independently prognostic (HR 5.019, 95% CI 1.212–20.789), and Hori 2020 [50] reported an independent WPOI association (HR 3.84, 95% CI 1.30–11.34), whereas the BD model was not retained for DFS.
Recurrence-free survival (RFS)	TB— Sakai [52]	No independent TB-specific RFS effect was retained. The primary prognostic model emphasized pathological DOI and tumor–stroma ratio rather than TB; therefore, the combined RFS result should not be attributed to TB.
Overall survival (OS)	TB/digital TB/TBI— Xie [36], Pedersen [40], Zheng [64], Alonso [67]	Higher TB was independently associated with poorer OS in Xie [36] (HR 5.582, 95% CI 1.227–25.381) and in Pedersen [40] using semi-automated digital tumor-bud count (HR 1.6 per tertile increase, 95% CI 1.1–2.2). In contrast, Zheng [64] found no significant TBG association with OS (*p* = 0.12), and Alonso [67] reported no significant OS difference by TBI (*p* = 0.219). Differences in TB assessment methods limit direct comparison.
DSS/OS with expanded WPOI-5	Expanded WPOI-5— Mohamed [55]	Expanded WPOI-5 and its components were associated with DSS and OS on univariable analysis. DSS could not be modeled multivariably because only 15 disease-specific deaths occurred. For OS, an alternative multivariable model including the composite WPOI-5 reported OR 7.57 (95% CI 3.67–15.60); when individual components were modeled instead, satellite nodules and extratumoral LVI remained independently associated. The expanded construct is not directly interchangeable with conventional satellite-based WPOI-5.
Composite histologic risk constructs	Arora [38], Sinha [41], Larson [48]	Survival associations derived from composite histologic models should not be attributed to TB or WPOI alone. Arora [38] reported a 5-year survival gradient across a composite ANLPS score but no marker-specific survival coefficient. In Sinha [41], WPOI alone was not associated with disease-related death and the adjusted composite high-risk result was not significant (*p* = 0.065). In Larson [48], ≥2 adverse features among WPOI-5/PNI/LVI predicted DSS (HR 6.5; *p* = 0.02), whereas WPOI-5 alone did not drive the survival effect.

Note: Survival endpoints are summarized according to the definitions used in the primary reports. DSS and CSS were considered within the same disease-specific mortality domain, whereas DFS and RFS were retained as distinct endpoints. PFS was not an eligible survival endpoint. Linked/partially overlapping Almangush, Hori, and Cork reports were not interpreted as independent replications. Detailed report-level Kaplan–Meier findings, effect estimates, confidence intervals, and overlap notes are provided in Supplementary Table S10. Abbreviations: ANLPS, composite histopathologic score; BD, budding–depth model; CI, confidence interval; CSS, cancer-specific survival; DFS, disease-free survival; DOI, depth of invasion; DSS, disease-specific survival; HR, hazard ratio; LVI, lymphovascular invasion; OR, odds ratio; OS, overall survival; PFS, progression-free survival; PNI, perineural invasion; POI, pattern of invasion; RFS, recurrence-free survival; TB, tumor budding; TBG, tumor budding grade; TBI, tumor budding index; WPOI, worst pattern of invasion.

## Data Availability

The data supporting this systematic review are provided in the article and Supplementary Materials.

## Supplementary Materials

The following supporting information can be downloaded at: https://www.mdpi.com/article/10.3390/jcm15207949/s1, PRISMA 2020 Checklist JCM REPAGINATED; Table S1. Search strategies for bibliographic databases; Table S2. Supplementary electronic platform searches; Table S3. Supplementary citation searching; Table S4. Final identification and deduplication accounting; Table S5. Characteristics of the 38 included reports; Table S6. Linked or partially overlapping cohorts and handling in the synthesis; Table S7. Histopathological assessment of tumor budding and worst pattern of invasion in the included reports; Table S8. Associations of tumor budding and worst pattern of invasion with cervical nodal outcomes; Table S9. Local and locoregional recurrence outcomes associated with tumor budding and worst pattern of invasion; Table S10. Survival outcomes associated with tumor budding and worst pattern of invasion; Table S11. Follow-up, surveillance, and adjuvant treatment in the included reports; Table S12. Final QUIPS risk-of-bias summary for the 38 included reports.
