# Supplementary material for: Prognostic Value of Tumor Budding and Worst Pattern of Invasion in Clinically Node-Negative Early-Stage Lip and Oral Cavity Squamous Cell Carcinoma: A Systematic Review
Source: J Clin Med. 2026 Oct 14;15(20):7949. doi: 10.3390/jcm15207949 (PMC13705793; doi:10.3390/jcm15207949)
Supplement: Supplementary file 1 [file jcm-15-07949-s001.zip › Supplementary Materials JCM.pdf]

Supplementary Materials

Systematic review of tumor budding (TB) and worst pattern of invasion (WPOI) in clinically node-negative early-stage lip and oral cavity squamous cell carcinoma

Literature eligibility cutoff: 25 April 2026. **Search verification:** 19–20 August 2026. Searches were rerun for completeness and reproducibility after removal of the lower publication-date restriction, and Scopus was added as an additional bibliographic database. The eligibility cutoff was retained; reports published online or in print as eligible full-text journal articles after 25 April 2026 were outside the scope of the review.

Search principle: The search strategies were designed to maximize sensitivity for studies evaluating TB and/or WPOI. Outcome terms were not mandatory search components because eligible prognostic studies could report cervical nodal disease, recurrence, DFS, RFS, DSS/CSS, OS, or other oncological outcomes without using uniform outcome terminology in titles or abstracts.

Source classification: PubMed and Scopus were treated as bibliographic databases. ScienceDirect and Semantic Scholar were retained as supplementary electronic search platforms rather than described as conventional bibliographic databases. Backward and forward citation searching was performed separately using Citationchaser.

Table S1. Search strategies for bibliographic databases.

Database	Search strategy	Date handling / verification date	Records identified
PubMed	(<br>(<br>"Mouth Neoplasms"[Mesh]<br>OR "oral cavity"[Title/Abstract]<br>OR oral[Title/Abstract]<br>OR mouth[Title/Abstract]<br>OR tongue[Title/Abstract]<br>OR lip[Title/Abstract]<br>OR "head and neck"[Title/Abstract]<br>)<br>AND<br>(<br>"Carcinoma, Squamous Cell"[Mesh]<br>OR "squamous cell carcinoma"[Title/Abstract]<br>OR "squamous carcinoma"[Title/Abstract]<br>OR OSCC[Title/Abstract]<br>OR HNSCC[Title/Abstract]<br>)<br>AND<br>(<br>"tumor budding"[Title/Abstract]<br>OR "tumour budding"[Title/Abstract]<br>OR "tumor bud"[Title/Abstract]<br>OR "tumour bud"[Title/Abstract]<br>OR "tumor buds"[Title/Abstract]<br>OR "tumour buds"[Title/Abstract]<br>OR WPOI[Title/Abstract]<br>OR "worst pattern of invasion"[Title/Abstract]<br>OR "worst-pattern of invasion"[Title/Abstract]<br>OR "pattern of invasion"[Title/Abstract]<br>)<br>)<br>NOT tuberculosis[Title/Abstract]	No lower date limit. Eligible full-text journal articles published online or in print after 25 April 2026 were outside the eligibility window and excluded during screening.<br>Verification: 19 August 2026.	n = 430
Scopus	TITLE-ABS-KEY(<br>("oral cavity" OR oral OR mouth OR tongue OR lip OR "head and neck")<br>AND<br>("squamous cell carcinoma" OR "squamous carcinoma" OR OSCC OR HNSCC)<br>AND<br>("tumor budding" OR "tumour budding" OR "tumor bud" OR "tumour bud"<br>OR "tumor buds" OR "tumour buds" OR WPOI<br>OR "worst pattern of invasion" OR "worst-pattern of invasion"<br>OR "pattern of invasion")	No lower date limit. PUBYEAR BEF 2027 retained all 2026 records; eligible full-text journal articles published online or in print after 25 April 2026 were outside the	n = 481

Database	Search strategy	Date handling / verification date	Records identified
	)<br>AND NOT TITLE-ABS-KEY(tuberculosis)<br>AND PUBYEAR BEF 2027	eligibility window and excluded during screening.<br>Verification: 20 August 2026.	

Table S2. Supplementary electronic platform searches.

Platform	Search strategy	Date handling / verification date	Records identified
ScienceDirect	Find articles with these terms:<br><br>(<br>(“oral squamous cell carcinoma”<br>OR “oral cavity squamous cell carcinoma”<br>OR “oral tongue squamous cell carcinoma”<br>OR “head and neck squamous cell carcinoma”<br>OR OSCC)<br>AND<br>(“tumor budding”<br>OR “tumour budding”<br>OR WPOI<br>OR “worst pattern of invasion”<br>OR “worst-pattern of invasion”)<br>)<br><br>Title, abstract or author-specified keywords:<br><br>(“tumor budding”<br>OR “tumour budding”<br>OR WPOI<br>OR “worst pattern of invasion”<br>OR “worst-pattern of invasion”)	No lower date limit. Exact 25 April 2026 filtering was unavailable; eligible full-text journal articles published online or in print after 25 April 2026 were outside the eligibility window and excluded during screening.<br>Verification: 19 August 2026.	N = 97
Semantic Scholar	(<br>(“oral cancer”<br>OR “oral squamous cell carcinoma”<br>OR “oral cavity squamous cell carcinoma”<br>OR “oral tongue squamous cell carcinoma”<br>OR “head and neck squamous cell carcinoma”<br>OR OSCC<br>OR HNSCC)<br>AND<br>(“tumor budding”<br>OR “tumour budding”<br>OR WPOI<br>OR “worst pattern of invasion”<br>OR “worst-pattern of invasion”<br>OR “pattern of invasion”)<br>)<br>NOT tuberculosis	No lower date limit. Exact end-date filtering was unavailable; eligible full-text journal articles published online or in print after 25 April 2026 were outside the eligibility window and excluded during screening.<br>Verification: 19 August 2026.	N = 250

Table S3. Supplementary citation searching.

Method	Source / approach	Search limits, procedure, and verification date	Records identified
Backward citation searching	Citationchaser	The 37 reports considered included at verification were used as seed articles. No lower date limit was applied. Citationchaser returned 1,137 reference links (810 unique records). Reports published online or in print as eligible full-text journal articles after 25 April 2026 were outside the review scope and excluded during screening.<br>Verification: 19 August 2026.	N = 810 unique records<br>(1,137 reference links)
Forward citation searching	Citationchaser	The same 37 seed reports were used. No lower date limit was applied. Citationchaser returned 1,771 citation links (965 unique records). Reports published online or in print as eligible full-text journal articles after 25 April 2026 were outside the review scope and excluded during screening.<br>Verification: 19 August 2026.	N = 965 unique records<br>(1,771 citation links)

Table S4. Final identification and deduplication accounting.

Source / stage	Records (n)	Final accounting
PubMed	430	Bibliographic database
Scopus	481	Bibliographic database
ScienceDirect	97	Supplementary electronic platform
Semantic Scholar	250	Supplementary electronic platform
Citationchaser—backward	810	Unique records
Citationchaser—forward	965	Unique records
Total records identified	3,033	Before cross-source deduplication
Duplicate records removed	1,202	Within- and cross-source bibliographic duplicates
Records remaining after deduplication	1,831	Records entering title/abstract screening

Search verification and documentation

- The lower publication-date boundary used in the initial search was removed during verification. The eligibility cutoff of 25 April 2026 was retained.
- The PubMed verification export contained 430 records and was used exactly as exported for deduplication and PRISMA accounting.
- A Scopus search was performed on 20 August 2026 as part of the search-verification process. The Scopus export contained 481 records; 442 were already represented in the master set and 39 were new unique records. None of the 39 new Scopus records met the final eligibility criteria, and the final included-report list therefore remained unchanged.
- ScienceDirect and Semantic Scholar were retained as supplementary electronic search platforms and were not presented as equivalents of PubMed or Scopus.
- Backward and forward citation tracking was repeated as part of search verification using the 37 reports considered included at that time. The final Citationchaser outputs were 810 unique backward-citation records and 965 unique forward-citation records.
- After cross-source deduplication, 1,831 unique records remained for screening. The final systematic review included 38 reports; linked or partially overlapping cohorts were handled separately to avoid double counting in synthesis.
- Outcome terms were not required in the search syntax because the eligibility criteria included occult/pathologic or delayed cervical nodal disease, local/regional/locoregional recurrence, DFS, RFS, DSS/CSS, and OS. Requiring a specific outcome vocabulary at the search stage could have reduced sensitivity.

Table S5. Characteristics of the 38 included reports.

No.	Report	Study design / recruitment	Eligible population, site and n	Clinical stage / TNM edition	Marker	Cervical management	Eligible outcome(s)	Linked cohort
1	Li, 2013	Multicenter retrospective validation cohort; recruitment NR	Stage I/II OCSCC; n=292 analyzed (299 characterized)	Stage I/II; TNM edition not stated	WPOI / histologic risk model	Primary surgery; neck management NR	Locoregional recurrence; DSS	—
2	Almangush, 2014	Multicenter cohort; 5 Finnish university hospitals	T1/T2 N0M0 OTSCC; n=233	T1/T2 N0M0; TNM edition not stated	TB + WPOI	Primary surgery; neck management NR	Disease-specific mortality / survival	A
3	Almangush, 2015a	Multicenter retrospective; 1979-2010	cT1-2 cN0 cM0 OTSCC; n=311 (Finland/Brazil)	cT1-2 cN0 cM0; TNM edition not stated	TB (BD model)	Primary surgery; neck management NR	Locoregional recurrence; OTSCC-related death	A
4	Almangush, 2015b	International multicenter cohort; recruitment NR	cT1-2N0 OTSCC; n=479 (Finland/Brazil/USA)	cT1-2N0; TNM edition not stated	WPOI + DOI	Primary surgery; neck management NR	Locoregional recurrence; disease-related mortality	A
5	Xie, 2015	Retrospective cohort; Jan 2001-Dec 2010	cT1/2N0M0 tongue SCC; n=195	cT1/2N0M0; UICC TNM, edition not stated	TB	Primary tumor resection; END in a subset; exact END/observation distribution NR	Cervical LNM; local recurrence; OS	—
6	Heerema, 2016	Retrospective cohort; 1997-2008	Low-stage OSCC; n=211	Low-stage; TNM edition not stated	Pattern of invasion / WPOI	Surgery; neck management NR	Locoregional recurrence-free survival; DSS	—
7	Arora, 2017	Retrospective analytical study; archived cases reviewed Oct-Dec 2016	cT1/2N0M0 OSCC; n=336	cT1/2N0M0; AJCC 8th edition (retrospective restaging)	TB + pattern of invasion	Prophylactic selective ND in 181/336; 155 initially observed, with delayed modified radical ND for nodal relapse in 40	Lymph-node metastasis; 5-year OS	—
8	Hori, 2017	Retrospective cohort; Sep 2000-Mar 2015	cT1/2N0M0 OTSCC; n=48	cT1/2N0M0; TNM edition not stated	TB + tumor depth	Primary partial glossectomy; observation; salvage ND for neck recurrence	Late/delayed LNM; regional control; OS	B
9	Pedersen, 2017	Consecutive early-stage cohort; source cohort n=253, digital analytic cohort n=222	cT1-2N0 OSCC; analytic n=222/253	cT1-2N0; UICC TNM 6th edition	TB / digital tumor-budding count	Sentinel-node biopsy in all source-cohort patients; N+ endpoint also included isolated later nodal recurrence	Composite nodal endpoint; OS	—
10	Sinha, 2018	Retrospective cohort; Jan 2001-Dec 2013	Stage I/II tongue or floor-of-mouth SCC; n=65	Stage I/II; AJCC 7th edition	WPOI / histologic risk model	Primary surgery; neck management NR	Recurrence; disease-related death / DSS	—
11	Sakata, 2018	Retrospective cohort; Jan 2001-Sep 2015	cT2N0 tongue SCC; n=97	cT2N0; AJCC 8th edition	TB	Transoral partial glossectomy alone; END and neoadjuvant therapy excluded; postoperative observation	Occult/delayed cervical metastasis; cause-specific survival	—
12	Shimizu, 2018	Retrospective cohort; Jan 2004-Dec 2013	cT1/2N0M0 OSCC; n=91	cT1/2N0M0; AJCC/UICC 7th edition	TB + WPOI / MOI	Transoral resection with or without END (12 END)	Regional metastasis; 5-year DFS	—
13	Yamanouchi, 2018	Observational cohort; Mar 2006-Apr 2013	Stage I/II tongue SCC; n=23	Stage I/II; UICC TNM 7th edition	TB + invasion mode	Glossectomy alone 19/23; concomitant ipsilateral SOHND 4/23	Secondary cervical LNM; DFS	—

No.	Report	Study design / recruitment	Eligible population, site and n	Clinical stage / TNM edition	Marker	Cervical management	Eligible outcome(s)	Linked cohort
14	Ebihara, 2019	Retrospective cohort; 2007-2015	cT1/2N0 tongue SCC; n=64	cT1/2N0; UICC/AJCC 7th edition (also reclassified to 8th for analysis)	TB	Wait-and-watch 42/64; END 22/64	Neck LNM; DSS	—
15	Yamakawa, 2019	Retrospective multi-institutional; Jan 2008-Dec 2014	cT1/2N0M0 tongue SCC; n=337	cT1/2N0M0; AJCC/UICC 7th edition	TB + adjacent invasive-front tissue	Primary resection without END; wait-and-see	Delayed neck metastasis; OS	—
16	Dourado, 2020	Retrospective cohort; 1998-2014	Eligible clinical Stage I/II OSCC subgroup n=103 (whole cohort n=254)	Clinical Stage I/II; TNM clinical stage 7th edition	TB + tumor–stroma ratio	Radical surgery; cervical management distribution NR	DFS; CSS	—
17	Larson, 2020	Retrospective cohort; 2000-2017	Early oral tongue SCC <=2 cm; n=100	Early T-stage; TNM edition not stated	WPOI-5 + adverse histology	Primary surgery; neck management variable/NR	Occult nodal positivity; 2-year LRR; DSS; OS	—
18	Parekh, 2020	Retrospective cohort; recruitment NR	pT1/T2N0 OCSCC; n=95	pT1/T2N0; TNM edition not stated	WPOI + TB / cell-nest size	Upfront resection + elective nodal dissection	DFS / recurrence-related outcome	—
19	Hori, 2020	Retrospective cohort; linked Kanagawa series	Clinical N0 early OTSCC; n=62	cN0 early OTSCC; AJCC/UICC 8th edition	WPOI + BD model	Primary surgery; surveillance-based cervical follow-up	Regional control; DFS / late nodal disease	B
20	Hori, 2021	Retrospective cohort; Sep 2000-Mar 2017	Early OTSCC; n=62	Early-stage; AJCC/UICC 8th edition	TB + tumor-infiltrating lymphocytes	Primary tumor excision; surveillance; ND for neck recurrence	Regional control; DFS	B
21	Sakai, 2022	Retrospective cohort; 2007-2017	cT1/2N0 OTSCC; n=70	cT1/2N0; TNM edition not stated	TB + tumor–stroma ratio	END n=16; no END n=54	Nodal metastasis; relapse / RFS	—
22	Salzano, 2022	Retrospective cohort; 2006-2021	cN0 T1-2 OTSCC; n=106	cN0 T1-2; TNM edition not stated	WPOI	SLNB	SLNB positivity	—
23	Lakhera, 2023	Prospective analytical observational cohort; from Apr 2018	Early-stage cN0 OSCC; n=100	Stage I/II cN0; AJCC 8th edition	WPOI	Transoral wide excision + ipsilateral selective neck dissection in all patients (systematic END)	Pathological / occult nodal involvement	—
24	Mohamed, 2023	Retrospective cohort; 2000-2020; Cork	pT1/2N0 OCSCC; n=160	pT1/2N0; TNM 7th edition	WPOI-5	Definitive primary surgery; ND not mandatory if cN0	DSS; OS; recurrence	C
25	Beute, 2023	Retrospective cohort; recruitment NR	Primary T1/T2 cN0 OCSCC; n=228	T1/T2 cN0; TNM edition not stated	WPOI-5	Primary resection + END	Occult cervical metastasis	—
26	Fonseca, 2023	Retrospective cohort; Jan 2016-Dec 2017	T1N0M0/T2N0M0 OSCC; n=207	T1N0M0 n=75; T2N0M0 n=132; AJCC 8th edition	WPOI / histologic risk score	Surgical resection; exact cervical management distribution NR	Locoregional recurrence	—
27	Kakuguchi, 2023	Retrospective cohort; Jan 2000-Apr 2022	Stage I/II tongue SCC; n=89	Stage I/II; TNM edition not stated	TB + WPOI / YK invasion pattern	Partial glossectomy only initially; no adjuvant therapy before CLNM	Cervical LNM / time-to-event outcome	—
28	Yamauchi, 2023	Retrospective cohort; recruitment NR	Early-stage tongue SCC; n=38	Early-stage; TNM edition not stated	WPOI	Primary resection without END; salvage ND for recurrence	Regional nodal recurrence; DSS	—
29	Yamazaki, 2023	Retrospective cohort; recruitment NR	Stage I/II tongue SCC; n=69	Stage I/II; TNM edition not stated	TB + WPOI-5+ Rac1	Partial glossectomy; postoperative observation for >2 years	Secondary / late LNM	—

No.	Report	Study design / recruitment	Eligible population, site and n	Clinical stage / TNM edition	Marker	Cervical management	Eligible outcome(s)	Linked cohort
30	Dey, 2024	Retrospective cohort; 2018-2023	Clinical Stage I/II cN0 OCSCC; n=102	Clinical Stage I/II; TNM edition not stated	WPOI + DOI / other histology	All underwent END	Occult nodal metastasis	—
31	Obayashi, 2024	Retrospective observational cohort; recruitment NR	cT1-2N0 tongue cancer; histopathologic subgroup n=61 (whole cohort n=102)	cT1-2N0; TNM edition not stated	TB + POI / tumor–stroma ratio	Primary surgery without END	Late cervical LNM	—
32	Singh, 2024	Prospective observational cohort; Mar 2020-Dec 2021	Clinical Stage I/II OSCC; n=237	Clinical Stage I/II; TNM edition not stated	TB + WPOI	Wide local excision + neck dissection	Pathological LNM	—
33	Zheng, 2024	Retrospective cohort; 2010-2020	cT1-2N0M0 buccal mucosa SCC; n=71	cT1-2N0M0; AJCC 8th edition	TB + DOI / mode of invasion	END 57/71; wait-and-watch 14/71	Occult cervical metastasis; LRRFS; OS	—
34	Hintze, 2025	Retrospective cohort from prospectively maintained database; 2000-2021; Cork	cT1/2N0 OCSCC; n=202 (pN0/pNx analytic set n=170)	AJCC 7th at cohort definition; restaged to AJCC 8th	WPOI-5 and components	END n=130; observation in remaining cN0 patients	Isolated regional recurrence; any regional recurrence; occult nodal disease	C
35	Kikuchi, 2025	Retrospective cohort; Apr 2009-Mar 2017	cT1-2N0M0 tongue SCC; n=66 (83 screened; 17 excluded for neoadjuvant therapy or unavailable clinical data)	cT1-2N0M0; AJCC 8th edition	TB + EMT markers	Primary tumor resection; prophylactic ND n=8; remaining patients observed; salvage/posterior ND in 13/17 late nodal metastases	Late nodal metastasis	—
36	Alonso, 2026	Retrospective longitudinal analytical cohort; Jan 2010-Dec 2019	cT1-2 cN0 oral tongue SCC; n=61	Stages I/II; AJCC 8th edition	TB / tumor budding index + IHC	Extensive primary tumor resection + elective neck dissection in all patients	Pathological cervical LNM; DFS; OS	—
37	Goyal, 2026	Prospective cross-sectional observational; Jun 2023-May 2024	T1/T2 cN0 OSCC; n=50	T1/T2 cN0; AJCC 8th edition	TB + WPOI + IHC markers	Pathological nodal status assessed; exact cervical surgical procedure not explicitly reported	Clinically occult / pathological nodal metastasis	—
38	Calabrese, 2026	Multicenter retrospective; surgery since Jan 2017; 30 Italian hospitals	cT1-2 cN0 OCSCC; n=1109 (source Results also report 1.9% cTis)	cT1-2 cN0; UICC TNM 8th edition	WPOI + DOI / other histology	END 682/1109; no END with clinical/ultrasound surveillance 427/1109	Occult nodal metastasis	—

Notes: NR = not reported or not directly verifiable in the primary report. N refers to the eligible early-stage subgroup when the full study cohort was broader. The TNM edition is recorded only when it was explicitly stated in the original report; it was not inferred from publication year. *Sinha et al.: The abstract states n=64, whereas the primary full-text tables consistently report n=65; the full-text denominator (n=65) was used for extraction and synthesis.

Table S6. Linked or partially overlapping cohorts and handling in the synthesis.

Cohort group	Linked reports	Evidence of overlap	Relationship between reports	Handling in the review
A —Almangush / multicenter early OTSCC	Almangush 2014; Almangush 2015a; Almangush 2015b	Shared author group and Finnish centers. The 2015a report explicitly states that 224 Finnish cases were part of the previously studied 233-patient Finnish cohort; the later international analysis expanded the source population.	Linked/partially overlapping multicenter cohorts; not three independent patient series.	All reports retained for distinct TB/WPOI analyses or outcomes. Overlapping participants are counted once in any independent-patient summary; for a duplicated endpoint, the most informative non-redundant analysis is used.
B—Hori / Kanagawa early OTSCC	Hori 2017; Hori 2020; Hori 2021	Same/related Kanagawa author group and institution, with overlapping early-OTSCC recruitment windows. Hori 2017 used a related earlier n=48 cohort; Hori 2020 and Hori 2021 used an essentially overlapping n=62 analytic cohort.	Serial/partially overlapping cohorts; Hori 2020 and Hori 2021 are not independent for shared endpoints.	Reports retained only for non-redundant prognostic analyses. Shared participants are not double counted, and overlapping outcomes are not treated as independent replications.
C—Cork early OCSCC	Mohamed 2023; Hintze 2025	Same Cork institutions and overlapping author group. Mohamed included n=160 patients from 2000-2020; Hintze included n=202 from an overlapping/extended 2000-2021 period with the same early-stage cT1/2N0 framework.	Extended/partially overlapping institutional cohort.	Mohamed contributes survival-oriented analyses and Hintze regional-recurrence/occult-node analyses. The reports are retained for distinct outcomes but not counted as independent cohorts in combined patient totals.

Note: Potential cohort overlap was assessed using author group, institution(s), recruitment period, sample size, and patient characteristics. Reports were considered linked when these features indicated complete or partial reuse of the same patient population. Linked reports were retained when they contributed distinct eligible outcomes or analyses, but overlapping participants were not double counted.

Table S7. Histopathological assessment of tumor budding and worst pattern of invasion in the included reports.

No.	Report	Marker	Definition / classification	Specimen / staining	Field selection / scoring method	Cut-off / analytical grouping	Observers / blinding / consensus
1	Li, 2013	WPOI / histologic risk model	Five-tier WPOI: 1 pushing border; 2 finger-like growth; 3 islands >15 cells; 4 islands <=15 cells; 5 satellites >=1 mm.	Surgical resection; H&E.	All available H&E slides reviewed for tumor–host interface.	Risk-model POI points: WPOI 1-3=0; WPOI 4=+1; WPOI 5=+3.	One expert pathologist (MBG), blinded to outcome.
2	Almangush, 2014	TB + WPOI	TB: isolated cell or cluster <5 cells in stroma at invasive front. WPOI from Brandwein-Gensler histologic risk model.	FFPE resection tissue; routine H&E.	Scan invasive front at x4; select hotspot; count buds at x20; highest count/case.	TB low <5 vs high >=5. WPOI low-risk cohesive vs high-risk small islands/satellites.	Three investigators; blinded to clinicopathologic data; pilot review with experienced H&N pathologist.
3	Almangush, 2015a	TB / BD model	TB as invasive-front isolated cells/small clusters <5 cells; combined with DOI in BD model.	H&E.	Detailed counting procedure referenced to prior work; not re-described in full in the report.	TB <5 vs >=5; DOI <4 vs >=4 mm; BD score 0-2.	NR.
4	Almangush, 2015b	WPOI + DOI	WPOI as invasive-front growth pattern; high-risk pattern characterized by small cell islands and/or satellites.	NR.	NR.	Lower-risk vs high-risk WPOI; DOI 4-mm threshold also analyzed.	NR.
5	Xie, 2015	TB	TB = isolated single cells or clusters of <5 cells ahead of the invasive front.	H&E; pan-cytokeratin IHC used to assist bud identification.	Initial scan at ×4 objective (×10 ocular); buds counted in a ×20 objective field at the tumor invasive front; maximum count retained.	Low <5 buds vs high ≥5 buds.	Two pathologists trained to a unified standard; independent assessment blinded to clinical data; consensus for disagreement. Interobserver κ=0.795; intraobserver κ=0.826 and 0.894.
6	Heerema, 2016	Pattern of invasion / WPOI	Five previously described pattern-of-invasion scoring approaches were compared.	NR.	Patterns scored at random and independently using the compared systems.	No single universal dichotomy; prognostic performance of several POI formulations compared.	Two independent observers; interobserver agreement assessed.
7	Arora, 2017	TB + pattern of invasion	TB = single tumor cell or cluster <5 cells in the stroma at the invasive front; POI scored on a four-tier invasive-front scale.	Routine histological sections (H&E morphology).	Single x20 field (0.785 mm²) at the invasive front for TB.	Low <5 vs high >=5 buds; POI types 1-4.	Two investigators evaluated all cases independently after joint training on 50 cases; discrepancies resolved by consensus. Overall interobserver kappa for the scoring procedure = 0.91.
8	Hori, 2017	TB	Bud = single tumor cell or cluster <5 cells at invasive front.	FFPE; H&E for TB; EvG and D2-40 used for vascular/lymphatic invasion.	Find highest-budding area at x4; count at x20.	Low <=4 vs high >=5 buds.	Review by author + experienced pathologist; pathologist blinded to patient course.
9	Pedersen, 2017	Digital TB count (DTBC)	Tumor bud operationalized as an IHC-positive tumor object with area <=950 micrometers² (approximately <=5 cells).	H&E used to select deepest-invasion block; IHC slide used for semi-automated digital analysis.	Whole selected slide; semi-automated image-analysis algorithm counted qualifying objects.	DTBC treated quantitatively and in model-derived risk strata; no simple manual 5-bud dichotomy.	Consultant H&N pathologist selected block; counting performed algorithmically.
10	Sinha, 2018	WPOI / histologic risk model	Brandwein-Gensler histologic risk model incorporating WPOI, PNI, and lymphocytic host response.	NR.	Tumor–host junction assessed using risk-model criteria.	WPOI contributes 0/1/3 points within composite low/intermediate/high-risk score.	NR.
11	Sakata, 2018	TB	TB = single cancer cell or cluster <5 cancer cells at the invasive front.	H&E and cytokeratin AE1/AE3 IHC; primary TB analysis used AE1/AE3.	Whole tumor scanned at x40 to select hotspot; buds counted at x200; highest count/sample retained.	ROC-derived cut-off >=4 buds for occult neck metastasis.	Two observers; consensus for discrepant assessments. AE1/AE3 interobserver kappa 0.774; intraobserver kappa 0.908 and 0.795 (H&E interobserver kappa 0.313).

No.	Report	Marker	Definition / classification	Specimen / staining	Field selection / scoring method	Cut-off / analytical grouping	Observers / blinding / consensus
12	Shimizu, 2018	TB + WPOI / MOI	TB = isolated/small tumor-cell clusters at invasive front. Conventional five-tier WPOI; WPOI-4 <=15-cell islands/strands/singles; WPOI-5 satellites >=1 mm.	H&E + pancytokeratin IHC.	Invasive-front hotspot assessment; representative scoring shown at x200.	TB 0-4 / 5-9 / >=10. WPOI analyzed by five-tier and higher-risk patterns.	NR.
13	Yamanouchi, 2018	TB + YK invasion pattern	TB evaluated at invasive front; YK classification used for invasion pattern.	H&E.	Initial x4 scan followed by bud count at x20.	G1 0-4, G2 5-9, G3 >=10; low = G1 (<5), high = G2/G3 (>=5). YK 1-3 vs 4C/4D.	Two independent observers (pathologist and oral surgeon), blinded to clinical outcome; reproducibility statistic NR.
14	Ebihara, 2019	TB	TB = single cancer cell or cluster <5 cancer cells at the invasive front.	Cytokeratin AE1/AE3 IHC (H&E alone considered insufficient for the final TBG evaluation).	Buds counted in the most intensive field using x20 objective.	TBG1 0-4, TBG2 5-9, TBG3 >=10; key multivariable comparison G1+G2 vs G3 (<10 vs >=10).	All specimens evaluated by the same oral pathologist; no interobserver statistic applicable.
15	Yamakawa, 2019	TB + adjacent invasive-front tissue / POI	TB = single cell or cluster <5 cells isolated at invasive front.	FFPE, 4-micrometer sections; H&E.	Scan at low magnification; identify area with most TB; count at x20.	Low=0; intermediate=1-4; high >=5 buds.	>2 pathologists; blinded to clinical data.
16	Dourado, 2020	TB	TB assessed at the invasive front; source describes buds as single cells or clusters of up to five cells; analytical positivity defined by ≥5 buds.	Routine H&E.	Invasive front scanned at low magnification; field with highest bud count assessed at ×200.	<5 vs ≥5 buds.	Single calibrated evaluator; 25 cases rescored after 4 weeks; intra-examiner κ=0.84 for TB.
17	Larson, 2020	WPOI-5	WPOI-5 recorded as an adverse invasive-front feature; exact operational wording incompletely reported in the primary report.	Histologic slide review; stain NR.	Retrospective pathology review.	WPOI-5 present vs absent.	NR.
18	Parekh, 2020	TB + WPOI	TB = single cells or clusters <5 cells. WPOI-4/5 evaluated at tumor–host interface.	Archived resection blocks; stain NR.	Bud count per x40 high-power field.	High TB >=3 buds/x40 HPF; emphasis on WPOI-4 and WPOI-5.	NR.
19	Hori, 2020	TB / BD model + WPOI	TB = isolated single cell or cluster <5 cells at the invasive front; WPOI assessed using the five-tier invasion-pattern framework.	FFPE resection specimens; H&E plus Elastica van Gieson, D2-40 and pan-cytokeratin AE1/AE3; TB assessed with pan-cytokeratin.	Low-power scan to identify the highest-budding area; buds counted at ×20 objective.	TB <5 vs >=5; BD positive = tumor depth >=3 mm plus TB >=5; WPOI analyzed separately.	Study author and experienced pathologist reviewed histology; slides/specimens were de-identified and the pathologist was blinded to clinical course.
20	Hori, 2021	TB	TB = isolated cell or cluster <5 cells.	Pan-cytokeratin IHC; other immune markers assessed separately.	Highest-budding area assessed at x20.	ITBCC-style: low 0-4; intermediate 5-9; high >=10.	Study author + experienced pathologist; slides de-identified; pathologist blinded.
21	Sakai, 2022	TB	TB grade assessed in cT1/2N0 OTSCC; standard small-cluster invasive-front concept.	H&E + cytokeratin AE1/AE3 IHC.	Hotspot-based assessment; exact field area NR.	Low <5 vs high >=5 buds.	NR.
22	Salzano, 2022	WPOI	Conventional five-tier WPOI classification.	Stain NR.	NR.	WPOI-1 to WPOI-5 analyzed as individual categories.	Number of observers and blinding NR.
23	Lakhera, 2023	WPOI	Five-tier WPOI; when multiple patterns were present, the highest/worst pattern was recorded.	Stain not explicitly reported.	Whole tumor–host interface reviewed for highest pattern.	WPOI 1-3 cohesive vs WPOI 4-5 invasive.	Two pathologists; blinded to clinical data; supplementary verification performed.
24	Mohamed, 2023	Expanded WPOI-5	WPOI-5 defined by satellite nodules and/or extratumoral PNI and/or extratumoral LVI.	Primary histology re-reviewed where appropriate; stain not explicitly stated.	Assessment specifically targeted WPOI-5 components.	WPOI-5 absent vs present; components also analyzed.	NR in extracted methodological text.
25	Beute, 2023	WPOI-5	WPOI-5 assessed in early OCSCC in intraoperative-consultation and permanent-section context.	Frozen/intraoperative material plus permanent histology.	Comparison of intraoperative and final WPOI-5 identification.	WPOI-5 present vs absent.	Pathology assessment; exact observer/blinding details NR in extracted text.

No.	Report	Marker	Definition / classification	Specimen / staining	Field selection / scoring method	Cut-off / analytical grouping	Observers / blinding / consensus
26	Fonseca, 2023	WPOI / histologic risk score	Five-tier Brandwein-Gensler WPOI: 1, pushing border; 2, finger-like growth; 3, islands >15 cells; 4, islands ≤15 cells; 5, satellites >1 mm from main tumor/closest satellite.	Routine H&E.	Archived resection slides reviewed and scored using the Brandwein-Gensler risk model.	WPOI 1-3 vs WPOI-4 vs WPOI-5 in the reported recurrence analysis.	Histopathological assessment performed by a pathologist blinded to clinical variables and patient outcomes.
27	Kakuguchi, 2023	TB + WPOI / YK	WPOI and TB evaluated; detailed original definitions not fully specified in report.	Stain NR.	Joint slide review.	TB <5 vs >=5; WPOI 2-3 vs 4-5 (WPOI-1 absent in cohort).	One oral pathologist + one oral surgeon; joint assessment; blinding NR.
28	Yamauchi, 2023	WPOI	WPOI classified according to established five-pattern invasive-front descriptions.	H&E.	Resection slides reviewed for WPOI.	WPOI 1-3 vs WPOI 4/5.	One head-and-neck pathologist; no access to individual clinical data.
29	Yamazaki, 2023	TB + WPOI-5+ Rac1	TB assessed as a histopathological feature; WPOI-5 assessed according to the AJCC 8th edition. Exact TB cell-count definition was not explicitly described in the primary report.	Surgical resection specimens. Rac1 expression assessed by immunohistochemistry; staining method specifically used for TB/WPOI assessment NR.	TB evaluated histopathologically; the analytical TB cut-off was determined by ROC analysis. WPOI-5 recorded as present/absent	TB <6 vs ≥6 buds; WPOI-5 negative vs positive.	Observer/blinding procedure for TB/WPOI NR. Rac1 immunohistochemistry was scored by two observers blinded to clinicopathological information
30	Dey, 2024	WPOI	Brandwein-Gensler: 1 broad pushing; 2 broad pushing fingers/large islands; 3 >15 cells; 4 <15 cells/singles/strands; 5 satellites separated by >=1 mm normal tissue.	Stain NR.	Highest invasive pattern recorded.	WPOI 1-3 cohesive; WPOI-4 infiltrative; WPOI-5 dispersive.	Observers/blinding NR.
31	Obayashi, 2024	TB + POI	TB = single cell or small tumor mass <5 cells isolated from invasive front. POI scale 1-5.	Stain not explicitly stated in extracted methods.	Low-power scan of invasive front; highest-budding area counted with x20 objective.	For the eligible late cervical LNM analysis: <3 vs >=3 buds; POI 1/2/3 vs 4/5. The source also used a separate >=7-bud threshold for distant-metastasis analysis, which was not retained as an eligible review outcome.	NR.
32	Singh, 2024	TB + WPOI	TB = isolated cell or cluster <5 cells. WPOI: 1 pushing; 2 finger-like; 3 >15 cells; 4 <=15 cells; 5 satellites >=1 mm.	FFPE; H&E + AE1/AE3 IHC.	Scan x4; hotspot; count at x20; highest count/slide.	ITBCC 0-4 / 5-9 / >=10 described, but analysis dichotomized <5 vs >=5. WPOI 1-3 vs 4-5.	NR.
33	Zheng, 2024	TB + MOI	TB = one cancer cell or a cluster of <5 cancer cells at the invasive tumor front.	Pan-cytokeratin IHC (AE1/AE3).	Immunostained slides scanned at ×4 objective (×10 ocular) to select highest-density area; buds counted at ×20 objective; highest count per slide retained.	TBG1 0-4; TBG2 5-9; TBG3 ≥10 buds.	Two experienced oral pathologists participated and were blinded to clinical course; the report subsequently states that all TBG specimens were evaluated by the same oral pathologist. Interobserver TBG reliability NR.
34	Hintze, 2025	Expanded WPOI-5	WPOI-5 = satellite nodules >=1 mm and/or extratumoral PNI/LVI; extratumoral defined >=1 mm beyond invasive front.	Re-review of histology after initial RCPath cohesive/non-cohesive assessment; stain NR.	Cases with non-cohesive invasion, PNI or LVI were re-reviewed to identify WPOI-5 components.	WPOI-5 absent vs present; components assessed individually.	Two pathologists using multiheaded microscope; consensus; blinding NR.

No.	Report	Marker	Definition / classification	Specimen / staining	Field selection / scoring method	Cut-off / analytical grouping	Observers / blinding / consensus
35	Kikuchi, 2025	TB + EMT markers	TB = isolated single cancer cell or a cluster of <5 cancer cells at the invasive front.	FFPE surgical resection specimens; H&E for histopathology. E-cadherin, vimentin and Ovol2 assessed separately by IHC.	Slides scanned with ×4 objective (×10 ocular) to select the highest-density budding area; buds counted at ×20 objective; highest count per slide retained.	ROC-derived cut-off: low TB <6 vs high TB >=6.	One oral pathologist diagnosed and assessed all histological sections; blinding/reproducibility assessment for TB not reported.
36	Alonso, 2026	TB / tumor-budding index (TBI)	TB defined as isolated tumor cells or small clusters at the invasive front; primary Methods classified low TBI as <5 buds/HPF and high TBI as >=5 buds/HPF.	Surgical resection specimens; H&E with subsequent cytokeratin AE1/AE3 IHC confirmation; E-cadherin and Ki-67 also evaluated.	TB quantified at the invasive tumor front; H&E classification followed by AE1/AE3 confirmation.	Low <5 vs high >=5 buds/HPF in Methods; Results contain a minor alternative <=5/>5 wording, which was not used to redefine the extraction.	Observer number/blinding and interobserver reproducibility for TBI not explicitly reported.
37	Goyal, 2026	TB + WPOI	TB = single cell or cluster <5 cells at invasive front. WPOI: 1 pushing, 2 finger-like, 3 >15-cell islands <1 mm, 4 <15-cell islands <1 mm, 5 satellites >1 mm.	10% formalin, FFPE, H&E; IHC separately for E-cadherin/cyclin D1.	Scan at x4 to identify hotspot; count TB at x20.	TB low <5 vs high >=5 buds/field. WPOI 1-4 vs WPOI-5.	Observer number/blinding NR.
38	Calabrese, 2026	WPOI	WPOI recorded as a five-tier histopathological variable; values available in 301/1109 patients.	Multicenter routine pathology data; staining not standardized/reported.	Center-reported histopathological data collected through REDCap; central slide re-review not reported.	WPOI 1-5 described; univariable logistic analysis used source-defined low-risk vs high-risk WPOI; exact low/high mapping not stated.	Observer number, blinding and consensus procedures NR.

Abbreviations: BD, budding–depth; DOI, depth of invasion; DTBC, digital tumor bud count; EMT, epithelial–mesenchymal transition; FFPE, formalin-fixed paraffin-embedded; H&E, hematoxylin and eosin; H&N, head and neck; HPF, high-power field; IHC, immunohistochemistry; LVI, lymphovascular invasion; MOI, mode of invasion; NR, not reported or not directly verifiable in the primary report; PNI, perineural invasion; POI, pattern of invasion; TB, tumor budding; TBI, tumor budding index; WPOI, worst pattern of invasion; YK, Yamamoto–Kohama.

Methodological details were transcribed as reported by the original authors; no common post hoc definition or threshold was imposed.

The expanded WPOI-5 used in the Cork reports (Mohamed and Hintze) incorporates extratumoral PNI and/or LVI in addition to satellite tumor nodules and is therefore not directly interchangeable with the conventional satellite-based five-tier WPOI definition.

Table S8. Associations of tumor budding and worst pattern of invasion with cervical nodal outcomes.

Report	Population / cervical pathway	Endpoint	Marker / comparison	Events or rates	Univariable association	Adjusted effect estimate	Statistical note / interpretation
**A. Pathological or occult nodal disease identified through initial/elective pathological neck assessment**							
Singh, 2024	Stage I/II OSCC, n=237; wide local excision + neck dissection	Pathological LNM	TB <5 vs >=5; WPOI-1–3 vs WPOI-4/5	Exact marker-specific event table reported in source; model based on n=237	Chi-square: TB p<0.001; WPOI p<0.001; DOI p=0.001; grade p=0.008	TB OR 30.8 (12.365–76.731), p<0.001; WPOI-4/5 OR 4.5 (1.853–11.305), p=0.001	Logistic regression; TB and WPOI were strong independent predictors.
Dey, 2024	Clinical Stage I/II cN0 OCSCC, n=102; all underwent END	Occult pN+	WPOI-1–3 vs IV vs V	Overall 26/102 (25.5%); node-positive within WPOI: 21.7% (1–3), 23.0% (IV), 62.5% (V)	Pearson chi-square p=0.043	NR for marker-specific adjusted model	Unadjusted categorical association; no marker-specific adjusted OR/CI reported.
Lakhera, 2023	Early cN0 OSCC, n=100; wide excision + ipsilateral selective ND	Pathological / occult nodal involvement	WPOI-1–3 vs WPOI-4/5	Node-positive: 9/43 (20.9%) for WPOI-1–3 vs 26/57 (45.6%) for WPOI-4/5; WPOI-5: 17/32 (53.1%)	Overall WPOI vs nodal positivity p=0.01; aggressive WPOI vs occult nodal metastasis p=0.019	NR	Chi-square-based analyses; confidence intervals not reported.
Beute, 2023	Primary pT1/T2 cN0 OCSCC, n=228; primary resection + END	Occult cervical metastasis	WPOI-5 vs WPOI-3/4; stratified by pT/DOI	pT1: WPOI-5 13/26 (50.0%) vs WPOI-3/4 3/53 (5.7%); pT2: 10/35 (28.6%) vs 23/114 (20.2%)	pT1 p<0.0001; pT2 not significant. Criterion WPOI-5 at any DOI OR DOI>=4 mm: p=0.017	No independent WPOI-specific adjusted effect retained	DOI >=4 mm was the dominant predictor; WPOI-5 effect was stage/DOI dependent.
Larson, 2020	Early OTSCC <=2 cm, n=100; surgical cohort with occult-node assessment	Occult nodal positivity	WPOI-5; DOI-stratified	No higher occult-metastasis rate attributable to WPOI-5 in either DOI stratum	Not significant within DOI strata	NR	Negative WPOI evidence for occult nodal positivity after DOI stratification.
Hintze, 2025 (C)	cT1/2N0 OCSCC, n=202; END n=130	Occult pN+ at END	Expanded WPOI-5 (satellite >=1 mm and/or extratumoral PNI/LVI)	Occult pN+ 32/130 (24.6%); WPOI-5: 12 pN0 vs 6 pN+	WPOI-5 p=0.38; satellite p=0.52; extratumoral LVI p=0.15; extratumoral PNI p=0.57	WPOI-5 not retained; LVI OR 3.6 (1.2–10.9), p=0.026	Expanded WPOI-5 definition; occult-node analysis negative for WPOI-5. Linked Cork cohort C.
Alonso, 2026	cT1–2 cN0 oral tongue SCC, n=61; all underwent elective neck dissection	Pathological cervical lymph-node metastasis	TBI low <5 vs high >=5 buds/HPF	pN+ 21/61 (34.4%). High TBI: 19/27 pN+ (70.4%); low TBI: 2/34 pN+ (5.9%).	TBI vs cervical metastasis p<0.001	TBI was the only independent predictor in binary logistic regression; 95% CI 1.19–116.68, p=0.035; point OR was not printed in the primary report.	Initial/elective pathological neck-assessment endpoint. The paper also states that 19/21 pN+ cases (90.5%) had high TBI; this was not misread as 90.5% of high-TBI cases being pN+.
**B. Sentinel lymph node biopsy positivity**							
Salzano, 2022	cN0 T1–2 OTSCC, n=106; all underwent SLNB	SLNB positivity	WPOI-I to WPOI-V	SLNB+ 35/106 (33.0%). Positivity within WPOI: I 2/9 (22.2%); II 5/29 (17.2%); III 7/32 (21.9%); IV 13/23 (56.5%); V 8/13 (61.5%)	Spearman Csp=0.342, p<0.001	No adjusted WPOI-specific effect reported	Distinct sentinel-node endpoint; not pooled with END-derived pN status or surveillance failure.
**C. Delayed/late cervical metastasis or regional nodal recurrence during surveillance/follow-up**							
Hori, 2017 (B)	cN0 early OTSCC, n=48; primary surgery without END	Late neck recurrence / LNM	High TB >=5 vs low <=4	Neck recurrence: 8/13 (62%) high TB vs 1/35 (3%) low TB	High TB p<0.001	Cox risk ratio 24.07 (2.27–254.89), p<0.01	Tumor depth >=3 mm + high TB: sensitivity 89%, specificity 95%, PPV 80%, NPV 97%. Linked Kanagawa cohort B.
Sakata, 2018	cT2N0 tongue SCC, n=97; transoral partial glossectomy alone; END excluded; postoperative observation	Occult/delayed cervical metastasis	TB <4 vs >=4 buds (ROC-derived)	Occult neck metastasis 16/97. TB>=4: 14/33 (42.4%) vs TB<4: 2/64 (3.1%).	TB p<0.001 univariable	Adjusted OR 9.014 (95% CI 1.563-89.075), p=0.012	Primary full-text Table 2; surveillance-defined nodal endpoint.

Report	Population / cervical pathway	Endpoint	Marker / comparison	Events or rates	Univariable association	Adjusted effect estimate	Statistical note / interpretation
Yamanouchi, 2018	Stage I/II tongue SCC, n=23; glossectomy alone 19/23 with cervical surveillance; concomitant ipsilateral SOHND 4/23	Secondary cervical LNM / locoregional recurrence with cervical LNM	Low-grade TB <5 vs high-grade TB >=5	Low-grade TB: 5 secondary cervical LNM + 1 LRR/CLM among 15 patients; high-grade TB: 5 secondary cervical LNM + 1 LRR/CLM among 8 patients.	No formal marker-specific statistical test for the nodal endpoint was reported.	NR	Descriptive cervical-event evidence only; the reported TB association was with DFS (log-rank p=0.047) and is summarized in Table S10.
Yamakawa, 2019	Early tongue SCC, n=337; primary resection without END / wait-and-see	Delayed neck metastasis	TB 0 vs 1–4 vs >=5 buds	DNM rates: 10.4% (0), 26.2% (1–4), 47.2% (>=5)	Intermediate p=0.0012; high p<0.0001 vs low	Intermediate HR 1.24 (0.59–2.61), p=0.5738; high HR 2.22 (1.15–4.30), p=0.0179	Multivariable Cox model; only high TB remained independently associated.
Hori, 2020 (B)	Clinical N0 early OTSCC, n=62; primary surgery; linked Kanagawa surveillance cohort	Regional control / late nodal failure	WPOI; BD model (depth >=3 mm + TB >=5)	5-year regional control 74%	WPOI and BD model significant on univariable analysis	WPOI HR 3.67 (95% CI 1.12–14.06), p<0.05; BD model HR 5.46 (1.08–27.52), p<0.05	Multivariable Cox model; linked cohort B and not an independent replication of Hori 2021. The primary Results text reports an upper 95% CI of 4.06 for WPOI, whereas Table 2 reports 14.06; the tabulated Cox-model estimate was retained.
Hori, 2021 (B)	Early OTSCC, n=62; primary surgery only; ND for neck recurrence	Regional control	Intermediate/high TB vs low TB	15 neck recurrences within 2 years	Budding associated with RC in model development	Budding score HR 17.91 (2.28–140.73), p=0.006	Cox proportional-hazards model; linked/overlapping Kanagawa cohort B, therefore not treated as independent replication.
Yamazaki, 2023	Stage I/II tongue SCC, n=69; partial glossectomy; postoperative observation for >2 years	Secondary lymph node metastasis (SLNM)	TB <6 (n=38) vs >=6 (n=31); WPOI-5 negative (n=60) vs positive (n=9)	Marker-specific SLNM event counts were not provided in the published Cox table.	Published Table 2: TB HR 2.76 (95% CI 0.94–8.09), p=0.06; WPOI-5 HR 2.85 (0.91–8.95), p=0.07.	Neither TB nor WPOI-5 was retained in the multivariable model; Rac1 was the only independent factor (HR 3.46, 95% CI 1.04–11.5; p=0.04).	Cox time-to-event analysis. The tabulated primary-report estimates are preferred: despite the abstract describing TB as significant in univariable analysis, the published Table 2 reports p=0.06 for TB.
Kakuguchi, 2023	Stage I/II tongue SCC, n=89; partial glossectomy initially; no chemo/RT until CLNM	Cervical LNM / nodal-metastasis-free survival	TB >=5 vs 0–4; WPOI-4/5 vs WPOI-2/3	CLNM 23/89 (25.8%); TB and WPOI distributions reported separately	Log-rank: TB >=5 p=0.009; WPOI-4/5 p=0.003	Neither TB nor WPOI retained in final Cox model; YK-4c and pDOI >5 mm strongest	The p=0.003 result is from a time-to-event log-rank analysis, not a crude comparison of WPOI proportions.
Yamauchi, 2023	Early tongue SCC, n=38; primary resection without END; salvage ND for recurrence	Regional nodal recurrence	WPOI-1–3 vs WPOI-4/5	The primary report states that recurrence was significantly higher with WPOI-4/5; the exact effect estimate was not directly verifiable and was therefore not retained.	Significant; exact p not retained without primary full-text verification	NR	Kept qualitative because the numerical effect estimate could not be directly verified in the primary report; no secondary numerical estimate was substituted.
Obayashi, 2024	cT1–2N0 tongue cancer; histopathologic subgroup n=61; no END	Late cervical LNM	TB <3 vs >=3; POI 1–3 vs 4/5	TB <3: 0/31; TB >=3: 15/30. POI 1–3: 2/26 vs POI 4/5: 13/35	TB p<0.0001; POI p=0.014	TB remained significant; reported numerical coefficient was extreme/unstable because one TB stratum had zero events	Zero-event separation makes the coefficient unstable; effect direction rather than magnitude is emphasized.
Hintze, 2025 (C)	Combined pN0/pNx n=170; mixed prior END/no END	Isolated regional recurrence; any regional recurrence	Expanded WPOI-5	Isolated RR 9/170 (5.3%); any RR 15/170 (8.8%)	Isolated RR: OR 5.95 (1.45–24.51), p=0.01; log-rank p=0.005. Any RR: OR 8.19 (2.84–23.60), p<0.0001	Isolated RR OR 5.21 (1.24–21.98); any RR OR 8.53 (1.76–41.41)	Mixed pN0/pNx cohort and expanded WPOI-5 definition; retained but interpreted separately. Linked Cork cohort C.
Kikuchi, 2025	cT1–2N0M0 tongue SCC, n=66; primary resection; prophylactic ND n=8	Late nodal metastasis	TB >=6 vs <6	17/66 (25.8%) late nodal metastases. TB >=6: 11/27; TB <6: 6/39. Median nodal recurrence time 10 months (range 2–26).	TB p=0.0256	Adjusted OR 3.78 (95% CI 1.19–12.10), p=0.025	Logistic regression; 8 prophylactic ND patients had no late nodal metastasis. Vimentin was also independently associated.
**D. Nodal endpoint with mixed or insufficiently described cervical ascertainment**							

Report	Population / cervical pathway	Endpoint	Marker / comparison	Events or rates	Univariable association	Adjusted effect estimate	Statistical note / interpretation
Xie, 2015	cT1/2N0M0 tongue SCC, n=195; primary tumor resection and/or END; exact END/observation distribution NR	Cervical lymph-node metastasis	Low vs high TB intensity	Nodal status available in 168: 111 negative and 57 positive; low/high TB among node-negative 59/52 and node-positive 19/38	TB intensity associated with nodal metastasis, p=0.015	No nodal OR/CI reported	Nodal status was verified from neck-dissection and regional-recurrence specimens; retained in mixed/insufficiently described cervical-pathway stratum.
Arora, 2017	cT1/2N0M0 OSCC, n=336; prophylactic selective ND 181, initial observation 155 with delayed ND after nodal relapse in 40	Nodal metastasis (mixed initial pathological + delayed nodal disease)	TB <5 vs >=5; POI types 1-4	Total nodal metastasis 92/336. TB low: 45/203 node+; TB high: 47/133 node+.	TB p=0.0089 univariable; POI p<0.0001	TB adjusted OR 1.28 (95% CI 1.09-2.61), p=0.039; POI OR 4.37 (1.07-15.34), p=0.007	Mixed END/surveillance ascertainment; primary full-text extraction.
Pedersen, 2017	cT1-2N0 OSCC; source cohort n=253 all underwent SNB; digital analytic cohort n=222	Composite N+ endpoint: metastasis identified during primary treatment plus isolated subsequent nodal recurrence	Semi-automated digital TB count	N+ definition combined initial nodal disease and later isolated nodal recurrence; exact subgroup event distribution not used as a pure SLNB endpoint	Prediction model AUC 0.83 (95% CI 0.78–0.89), p<0.001	10-fold cross-validation AUC 0.79 (0.73–0.85)	Sentinel-node procedure was universal, but the modeled N+ endpoint was composite; therefore kept separate from pure SLNB positivity.
Ebihara, 2019	cT1/2N0 tongue SCC, n=64; wait-and-watch 42, END 22	Neck lymph-node metastasis (mixed END + surveillance)	TBG G1/G2 vs G3 (G3 >=10 buds)	Pathological NLM ultimately confirmed in 21/64 (33%).	TBG associated with NLM; G1+2 vs G3 p<0.001	Adjusted OR 9.55 (95% CI 1.80-50.8), p=0.008	Primary full-text Table 3; mixed END/WW pathway.
Shimizu, 2018	cT1/2N0M0 OSCC, n=91; 12 END, otherwise surveillance	Regional metastasis (3 detected by END; 19 delayed)	TB; mode/WPOI-related invasion	22 regional metastases total	TB p<0.01; mode of invasion p<0.01; LVI p=0.04	Authors report 3.05 (95% CI 0.29–5.30), p<0.01, labelled HR	Internal source inconsistency retained: model is described as logistic regression, estimate is labelled HR, and stated CI crosses 1 despite p<0.01.
Sakai, 2022	cT1/2N0 OTSCC, n=70; END n=16, no END n=54	NLM during follow-up	TB grade	NLM n=35	TB p=0.008	TB not retained; pDOI p=0.01 and TSR p=0.02 retained	Mixed cervical management prevents classification as purely pathologic or delayed nodal endpoint.
Zheng, 2024	cT1-2N0M0 buccal mucosa SCC, n=71; END 57, wait-and-watch 14	Occult cervical metastasis	TBG1 vs TBG2 vs TBG3	20/70 evaluable (28.6%): 14 pN+ after END plus 6 cervical metastases in WW group; one patient lost to follow-up	Overall TBG association with CM in univariable logistic analysis, p=0.014	TBG2 vs TBG1 OR 6.08 (1.50–24.62), p=0.011; TBG3 vs TBG1 OR 7.13 (0.82–61.60), p=0.074; trend p=0.013	Adjusted for age, sex, T stage, neck policy and tumor differentiation. Mixed pathological/surveillance ascertainment.
Calabrese, 2026	cT1-2 cN0 OCSCC, n=1109; END 682, no-END surveillance 427	Occult nodal metastasis	Source-defined high-risk vs low-risk WPOI	Among END patients, 193/682 (28.3%) were pN+; non-END patients were assessed by clinical/instrumental follow-up, with pathological lymphadenopathy within 12 months classified as occult disease	High-risk WPOI OR 4.85 (2.95–8.07), p<0.001 in univariable logistic regression	WPOI not entered into the final multivariable model because it was available in only 301/1109 patients	Mixed ascertainment by pathological END status or 12-month surveillance. The abstract denominator “193 (28%) of 1109” reflects the END subgroup percentage, not 193/1109.
Goyal, 2026	T1/T2 cN0 OSCC, n=50; pathological nodal status reported; exact cervical surgical procedure not explicitly stated	Pathological / occult nodal metastasis	TB <5 vs >=5; WPOI 1–4 vs 5	LN+ 24, LN- 26. TB>=5: 12/24 (50.0%) node+ vs 6/26 (23.1%) node-. WPOI-5: 7/24 (29.2%) vs 2/26 (7.7%)	TB p=0.04; WPOI p=0.04	No adjusted marker model reported	Pathological nodal endpoint retained, but exact cervical surgical procedure remains legitimately NR in the primary Methods.

Categories reflect the clinical method by which cervical nodal disease was ascertained, not methodological quality: A, initial/elective pathological neck assessment; B, SLNB; C, delayed/late metastasis or regional recurrence during follow-up; D, mixed or insufficiently described ascertainment.

Abbreviations: AUC/AUROC, area under the receiver operating characteristic curve; BD, budding–depth; CI, confidence interval; CLNM, cervical lymph-node metastasis; DNM, delayed neck metastasis; DOI, depth of invasion; END, elective neck dissection; HR, hazard ratio; LNM, lymph-node metastasis; LVI, lymphovascular invasion; NR, not reported/not numerically available; OR, odds ratio; OCSCC, oral cavity squamous cell carcinoma; OTSCC, oral tongue squamous cell carcinoma; pN+, pathologically node-

positive; PNI, perineural invasion; PPV/NPV, positive/negative predictive value; RC, regional control; RR, regional recurrence; SLNB, sentinel lymph-node biopsy; TB, tumor budding; TSR, tumor–stroma ratio; WPOI, worst pattern of invasion; YK, Yamamoto–Kohama.

Effect measures and statistical labels are reproduced as reported by the primary authors. Where a primary source contained an internal numerical or labeling inconsistency, the inconsistency is explicitly flagged rather than silently corrected.

Linked reports from the Kanagawa cohort (B) and Cork cohort (C) were not interpreted as independent replications of the same endpoint. The WPOI-5 definition used in the Cork reports includes extratumoral PNI/LVI in addition to satellite nodules and is not directly interchangeable with conventional satellite-based WPOI-5.

Table S9. Local and locoregional recurrence outcomes associated with tumor budding and worst pattern of invasion.

Report	Population / endpoint	Marker / comparison	Events / crude data	Univariable / crude association	Adjusted / time-to-event association	Interpretation / overlap
Li, 2013	Stage I/II OCSCC; n=292 analyzed; LRR	WPOI; cut-points WPOI-4 and WPOI-5	56 LRR among T1/T2N0 patients (30 T1N0; 26 T2N0). WPOI-5: reported LRR probability 42%.	WPOI associated with LRR (p=0.0002).	WPOI-4 cut-point: HR 3.63 (95% CI 1.56–8.47), p=0.0029. WPOI-5 cut-point: HR 2.55 (1.48–4.41), p=0.0008.	Strong adjusted evidence for conventional WPOI.
Almangush, 2015a	cT1–2 cN0 cM0 OTSCC; n=311; LRR	BD score combining TB ≥5 and DOI ≥4 mm	NR.	High BD score associated with LRR.	High-risk BD score independently predicted LRR, p=0.033; no recurrence-specific HR was retained.	Linked cohort A; not an independent replication of other Almangush reports.
Almangush, 2015b	cT1–2N0 OTSCC; n=479; LRR	High-risk WPOI (small islands/satellites); DOI ≥4 mm	NR.	Cox models evaluated WPOI and DOI.	WPOI: HR 1.46 (95% CI 0.95–2.25). DOI ≥4 mm: HR 1.67 (1.07–2.60).	Linked cohort A. WPOI CI includes 1; adjusted WPOI effect on LRR was not statistically conclusive.
Xie, 2015	cT1/2N0M0 tongue SCC; n=195; local relapse	Low vs high TB intensity	Table 1: 22 local relapses overall; 3 low-TB and 19 high-TB.	TB intensity associated with local relapse, p=0.001.	No recurrence-specific adjusted effect estimate reported; Cox model addressed OS/prognosis.	Primary Table 1 retained. The narrative follow-up subsection reports a smaller follow-up subset, so the table-level full-cohort recurrence data were used for this association.
Heerema, 2016	Low-stage OSCC; n=211; LRRFS	Predominant POI; summed predominant + worst POI	Median follow-up 64 months.	Both scoring approaches prognostic for LRRFS.	Predominant POI: HR 2.1, p=0.033. Summed predominant + worst POI: HR 2.2, p=0.024 (multivariable Cox).	POI scoring rather than a single WPOI cut-off; interobserver κ 0.52–0.58.
Sinha, 2018	Stage I/II tongue/floor-of-mouth SCC; 65 evaluable in full text (abstract n=64); locoregional recurrence	Brandwein–Gensler WPOI within composite histologic risk model	LRR in 20/65 (31%).	Higher WPOI score associated with recurrence, p=0.032; WPOI-5/multifocality not associated, p=0.524. High composite risk category p<0.001.	Composite high-risk category vs low/intermediate: adjusted OR 12.4, p=0.001 (controlled for margins and T stage).	Adjusted effect is for the composite risk model, not WPOI alone.
Dourado, 2020	OSCC; clinical Stage I/II subgroup n=103; DFS	TB ≥5; TSR; combined TSR/TB model	Early-stage-specific recurrence count NR; DFS was defined as time to first local, regional or distant recurrence or last follow-up.	TB showed only a tendency toward poorer DFS: HR 1.98 (95% CI 0.92–4.27), p=0.07.	TB was not an independent DFS predictor; TSR HR 2.56 (1.18–5.55), p=0.017 and high-risk combined model HR 1.89 (1.16–3.10), p=0.01 were independently associated with DFS.	Corrects the abstract-level overstatement of recurrence significance for TB; primary Results/Discussion show p=0.07.
Larson, 2020	Small (≤2 cm) OTSCC; n=100; 2-year LRR	WPOI-5; PNI; LVI; stratified by DOI ≤4 vs >4 mm	WPOI-5 alone did not increase LRR within either DOI stratum.	No significant LRR difference for WPOI-5 alone.	Only ≥2 adverse features among WPOI-5/PNI/LVI predicted LRR: OR 5.7, p=0.01.	Negative evidence for an independent WPOI-5 effect; positive result belongs to a composite feature count.
Mohamed, 2023	T1/T2N0 OCSCC; n=160; LRRFS	Expanded WPOI-5 = satellites ≥1 mm and/or extratumoral PNI/LVI	32/160 recurrent: 25 local (8 also regional), 6 isolated regional, 1 isolated distant.	WPOI-5 OR 6.77 (95% CI 2.89–15.84), p<0.0001. Satellite nodules OR 6.53; extratumoral PNI OR 8.01; extratumoral LVI OR 23.46.	LRR multivariable: extratumoral LVI OR 13.49 (3.13–56.99), p=0.0004; extratumoral PNI OR 4.65 (1.17–18.54), p=0.03; any PNI OR 2.92 (1.23–6.93), p=0.02.	Linked Cork cohort C. Expanded WPOI-5 is not interchangeable with conventional satellite-only WPOI-5; composite WPOI-5 not retained with its overlapping components.
Fonseca, 2023	T1N0M0/T2N0M0 OSCC; n=207; LRR = initial site and/or regional nodes	WPOI 1-3 vs WPOI-4 vs WPOI-5	113 recurrences: WPOI 1-3, 45/113; WPOI-4, 67/91; WPOI-5, 1/3.	Overall WPOI category associated with LRR, p<0.00001 (log-rank).	No adjusted WPOI-specific effect estimate reported.	Only three patients had WPOI-5; the overall category association therefore cannot establish a WPOI-5-specific recurrence effect. The last two source-table columns are labelled as percentages but behave arithmetically as counts and were not used for this inference.

Report	Population / endpoint	Marker / comparison	Events / crude data	Univariable / crude association	Adjusted / time-to-event association	Interpretation / overlap
Zheng, 2024	cT1-2N0M0 buccal mucosa SCC; n=71; LRRFS	TBG1 vs TBG2 vs TBG3	10/71 local or regional recurrences.	Kaplan–Meier log-rank by TBG p=0.04; Table 2 univariable Cox overall TBG p=0.242.	Table 3: TBG2 vs TBG1 HR 1.01 (0.14–7.54), p=0.992; TBG3 vs TBG1 HR 12.38 (1.58–96.75), p=0.016; trend p=0.062.	Primary Table 3 was preferentially retained because the abstract/narrative reports discrepant HR 15.03 and trend p=0.03. Source-level inconsistency is documented explicitly.

Abbreviations: BD, budding–depth; CI, confidence interval; DFS, disease-free survival; DOI, depth of invasion; HR, hazard ratio; LRR, locoregional recurrence; LRRFS, locoregional recurrence-free survival; LVI, lymphovascular invasion; NR, not reported; OCSCC, oral cavity squamous cell carcinoma; OR, odds ratio; OTSCC, oral tongue squamous cell carcinoma; PNI, perineural invasion; POI, pattern of invasion; TB, tumor budding; TSR, tumor–stroma ratio; WPOI, worst pattern of invasion.

Regional-only or delayed cervical nodal recurrences already synthesized as cervical nodal outcomes are not duplicated in this table.

The Almangush reports belong to linked/partially overlapping cohorts (Table S6) and are not interpreted as independent replications. Mohamed et al. belongs to the linked Cork cohort and used an expanded WPOI-5 definition incorporating extratumoral PNI/LVI.

For Fonseca et al., only three patients had WPOI-5 and one recurred; therefore, the significant overall WPOI association should not be interpreted as evidence of a WPOI-5-specific recurrence effect.

Table S10. Survival outcomes associated with tumor budding and worst pattern of invasion.

Report	Population / endpoint	Marker / comparison	Kaplan–Meier / crude finding	Adjusted / time-to-event estimate	Statistical status	Interpretation / overlap
Li, 2013	Stage I/II OCSCC; n=292 analyzed; DSS	WPOI-5 vs WPOI-1–4	18 disease-specific deaths among T1/T2N0 patients.	Adjusted DSS HR 6.34 (95% CI 2.50–16.09), p=0.0001.	Multivariable Cox; significant	Strong conventional WPOI-5 evidence; separate composite Risk Model HR 9.16 (2.65–31.66).
Almangush, 2014	T1/T2N0M0 OTSCC; n=233; mortality due to OTSCC / DSS	TB ≥5 vs <5; high- vs low-risk WPOI	55 deaths due to OTSCC; log-rank associations reported for TB and WPOI.	TB HR 2.04 (1.17–3.55), p=0.01; WPOI HR 4.47 (1.59–12.51), p=0.004.	Adjusted Cox; both significant	Linked cohort A; adjusted for age, sex, grade, and center.
Almangush, 2015a	cT1–2 cN0 cM0 OTSCC; n=311; OTSCC-related death	High-risk BD score (TB ≥5 + DOI ≥4 mm)	High-risk BD score associated with cancer-related death.	Independent association, p<0.001; coefficient NR.	Multivariable; significant	Linked cohort A; composite BD model, not TB alone.
Almangush, 2015b	cT1–2N0 OTSCC; n=479; disease-specific mortality	Invasive/high-risk WPOI vs cohesive	89/479 died from OTSCC in the full analyzed cohort.	WPOI HR 2.34 (95% CI 1.26–4.32).	Multivariable Cox; significant	Linked cohort A; related/expanded population, not an independent replication.
Xie, 2015	cT1/2N0M0 tongue SCC; n=195; OS follow-up available for n=106	High vs low TB intensity	Kaplan–Meier OS significantly worse with high TB (p<0.001).	High TB adjusted HR 5.582 (95% CI 1.227–25.381), p=0.026.	Adjusted Cox; significant	Estimate extracted directly from primary Table 2. The narrative text juxtaposes HRs for TB and local relapse ambiguously; Table 2 was used as the authoritative source.
Heerema, 2016	Low-stage OSCC; n=211; DSS	Predominant POI; summed predominant + worst POI	Pattern of invasion prognostic for DSS.	Predominant POI HR 2.3, p=0.032; summed predominant+worst POI HR 2.1, p=0.044.	Multivariable Cox; significant	POI scoring systems, not a single conventional WPOI dichotomy; moderate interobserver reproducibility.
Pedersen, 2017	Early-stage OSCC; n=222; OS	Digital tumor bud count (DTBC), per tertile increase	Higher DTBC associated with poorer OS.	OS HR 1.6 (1.1–2.2), p=0.01.	Multivariable Cox; significant	Semi-automated whole-slide/digital budding metric; not directly interchangeable with manual bud cut-offs.
Arora, 2017	cT1/2N0M0 OCSCC; n=336; 5-y survival	Composite ANLPS histopathologic score including invasive-front variables	5-y survival: low score 93%, moderate 67%, high ≥17: 39%.	Marker-specific TB/WPOI survival coefficient not reported separately.	Composite model	Survival gradient cannot be attributed specifically to TB or WPOI.
Sakata, 2018	cT2N0 tongue SCC; n=97; DSS	TB score ≥4 vs <4	TB >=4 was strongly associated with worse cause-specific survival in univariable analysis; occult neck metastasis was the only independent prognostic factor in the final model.	Primary Table 3: TB HR 9.605 (95% CI 2.473-63.023), p<0.001 univariable; adjusted HR 3.114 (0.520-32.052), p=0.232.	Adjusted TB effect not significant	Direct primary full-text extraction; no secondary estimate used.
Shimizu, 2018	cT1/2N0M0 OSCC; n=91; 5-y DFS	TB low/intermediate/high; WPOI-5 vs WPOI-1–4	5-y DFS TB: 65.0% / 37.5% / 23.1%, p<0.01. WPOI-1–4 58.4% vs WPOI-5 33.3%, p=0.03.	TB intensity HR 2.18 (95% CI 1.49–3.20), p<0.01.	Final stepwise Cox: TB significant	WPOI was associated in KM analysis but not retained in final Cox model. Primary Results/Table estimate retained for TB; the abstract reports HR 2.19 (95% CI 1.51–3.18).
Yamanouchi, 2018	Stage I/II tongue SCC; n=23; DFS	TB <5 vs ≥5; low vs high YK invasion pattern	DFS significantly longer with low-grade TB (p=0.047); YK low vs high p=0.017.	No adjusted HR reported.	Log-rank / group comparison; significant	Small cohort; no effect estimate with CI.
Sinha, 2018	Stage I/II tongue/floor-of-mouth SCC; 65 evaluable; disease-related death	WPOI score; WPOI-5/multifocality; composite histologic risk category	11/65 disease-related deaths. WPOI alone p=0.177; WPOI-5/multifocality p=0.261. Composite high-risk category p=0.047.	Composite high-risk category adjusted OR 5.55, p=0.065.	WPOI-alone null; composite adjusted result not significant	Do not attribute the composite risk-model mortality signal to WPOI alone.
Ebihara, 2019	cT1/2N0 tongue SCC; n=64; DSS	Tumor budding grade 3 (TBG3)	TBG significantly associated with DSS in univariable analysis.	TBG3 HR 4.41 (95% CI 1.34–14.5), p=0.02.	Multivariable Cox; significant	Primary Table 3; direct TB-specific multivariable DSS estimate.

Report	Population / endpoint	Marker / comparison	Kaplan–Meier / crude finding	Adjusted / time-to-event estimate	Statistical status	Interpretation / overlap
Dourado, 2020	OSCC; clinical Stage I/II subgroup n=103; CSS and DFS	TB ≥5; TSR; combined TSR/TB model	Early-stage univariable: TB associated with CSS (p=0.001) but not significantly with DFS (p=0.07; HR 1.98, 95% CI 0.92–4.27).	CSS: TB HR 3.03 (1.30–7.07), p=0.01. DFS: TB not retained as an independent predictor; TSR HR 2.56 (1.18–5.55), p=0.017 and high-risk combined model HR 1.89 (1.16–3.10), p=0.01.	Adjusted CSS positive; DFS TB negative/non-significant	Primary Results and Discussion used rather than the abstract wording, which overstates the early-stage recurrence association.
Larson, 2020	Small (≤2 cm) OTSCC; n=100; DSS and OS	WPOI-5 alone; count of adverse features WPOI-5/PNI/LVI	WPOI-5 alone did not worsen OS within DOI ≤4 mm stratum; no single adverse feature drove survival.	≥2 adverse features: DSS HR 6.5, p=0.02.	Composite feature count significant	Adjusted survival result is not a WPOI-5-alone effect.
Parekh, 2020	pT1/T2N0 OCSCC; n=95; DFS	WPOI-4/5; WPOI-5 alone; high TB ≥3/×40 HPF	Univariable: WPOI-4/5 p=0.033; high TB p=0.021; WPOI-5 alone reported as non-significant.	WPOI-4 HR 5.019 (95% CI 1.212–20.789), p=0.026.	Multivariable Cox: WPOI-4 significant	Supports WPOI-4 rather than a WPOI-5-specific DFS effect.
Hori, 2020	Clinical N0 early OTSCC; n=62; 5-y DFS	WPOI; BD model	5-y DFS 65%; WPOI and BD model significant in univariable analysis.	WPOI HR 3.84 (95% CI 1.30–11.34), p<0.05; BD model not retained as an independent DFS predictor.	Multivariable Cox; WPOI significant	Linked cohort B; direct primary full-text estimate.
Hori, 2021	Early OTSCC; n=62; 5-y DFS	Intermediate/high TB vs low TB	5-y DFS 65%; budding significant in univariable analysis (p<0.01).	TB HR 1.95 (95% CI 0.79–4.84), p=0.15.	Adjusted Cox; not significant	Linked cohort B, substantially overlapping Hori 2020; CD163+ macrophages, not TB, independently predicted DFS.
Sakai, 2022	cT1/2N0 OTSCC; n=70; RFS	TB grade; tumor–stroma ratio; pDOI	5-y RFS was 78% for pDOI≤5/stroma-poor vs 33% for pDOI>5/stroma-rich.	No independent TB-specific RFS estimate was retained.	TB not established as independent RFS predictor	Primary prognostic model emphasized pDOI/TSR; avoid assigning combined RFS result to TB.
Mohamed, 2023	T1/T2N0 OCSCC; n=160; DSS	Expanded WPOI-5 (satellites and/or extratumoral PNI/LVI)	15 index-cancer deaths. WPOI-5 and all components significantly associated with DSS on univariable analysis.	DSS not entered into multivariable model because of the small number of events.	Univariable significant; no adjusted DSS estimate	Linked Cork cohort C; expanded WPOI-5 differs from conventional satellite-only definition.
Mohamed, 2023	T1/T2N0 OCSCC; n=160; OS	Expanded WPOI-5	WPOI-5 and all three components significantly associated with OS univariably.	Alternative multivariable model including composite WPOI-5: reported OR 7.57 (95% CI 3.67–15.60).	Multivariable; significant	Authors reported OR, not HR; preserved without conversion. Linked cohort C.
Yamauchi, 2023	Early-stage tongue SCC; n=38; 5-y DSS	WPOI-1–3 vs WPOI-4/5	5-y DSS 100% vs 56%, p=0.0001; 4 disease-specific deaths overall.	No adjusted HR reported.	Kaplan–Meier/log-rank; significant	Only one patient had WPOI-5; effect represents grouped WPOI-4/5, not WPOI-5 alone.
Zheng, 2024	cT1-2N0M0 buccal mucosa SCC; n=71; OS	TBG; DOI; MOI	No significant association of TBG, DOI or MOI with OS; TBG log-rank p=0.12.	No significant marker-specific adjusted OS association reported.	Negative	Primary report supports a recurrence-specific, not OS, prognostic signal for the highest TBG category.
Alonso, 2026 (online 2025)	cT1–2 cN0 oral tongue SCC; n=61; DFS and OS	TBI low vs high	Time to locoregional recurrence: 93.9±7.0 vs 74.8±10.2 months, log-rank p=0.076. OS: 93.2±7.6 vs 83.9±9.3 months, p=0.219.	No significant adjusted survival effect reported.	Negative for DFS/OS	All patients underwent END; nodal prognostic signal did not translate into statistically significant DFS or OS differences.

Abbreviations: BD, budding–depth; CI, confidence interval; CSS, cancer-specific survival; DFS, disease-free survival; DOI, depth of invasion; DSS, disease-specific survival; DTBC, digital tumor bud count; HR, hazard ratio; LVI, lymphovascular invasion; NR, not reported; OCSCC, oral cavity squamous cell carcinoma; OR, odds ratio; OS, overall survival; OTSCC, oral tongue squamous cell carcinoma; PNI, perineural invasion; POI, pattern of invasion; RFS, recurrence-free survival; TB, tumor budding; TBG, tumor budding grade; TBI, tumor budding index; TSR, tumor–stroma ratio; WPOI, worst pattern of invasion.

Eligible survival endpoints were DFS, RFS, DSS/CSS, and OS. DSS and CSS were treated within the same disease-specific survival domain while preserving the terminology used by the original authors; DFS and RFS were not assumed to be interchangeable when study definitions differed. PFS was not treated as an eligible survival endpoint. When available, adjusted time-to-event estimates were preferred over crude proportions or final event counts.

Linked/partially overlapping reports (Almangush cohort A; Hori cohort B; Cork cohort C) are retained for non-redundant analyses but are not interpreted as independent replications.

For Mohamed et al., the original authors report odds ratios for multivariable OS models; values are reproduced as reported and were not converted to hazard ratios.

Table S11. Follow-up, surveillance, and adjuvant treatment in the included reports.

Report	Follow-up duration / timing	Postoperative / cervical surveillance and salvage strategy	Adjuvant treatment	Relevant notes
Li, 2013	NR	Primary surgery; cervical surveillance strategy NR.	NR	Exact follow-up duration and schedule were not directly verifiable and are reported as NR.
Almangush, 2014	NR	Primary surgery; cervical management/surveillance NR.	NR	Linked/partially overlapping cohort A.
Almangush, 2015a	NR	Primary surgery; cervical management/surveillance NR.	NR	Linked/partially overlapping cohort A; recruitment 1979-2010.
Almangush, 2015b	NR	Primary surgery; cervical management/surveillance NR.	NR	Linked/partially overlapping cohort A.
Xie, 2015	NR	Cervical ascertainment/follow-up pathway insufficiently detailed for END-versus-surveillance classification.	NR	Exact follow-up schedule unavailable.
Heerema, 2016	Median follow-up 64 months.	Surgical treatment; cervical surveillance strategy NR.	NR	Long-term locoregional and disease-specific outcomes were available.
Arora, 2017	Mean follow-up 72.4 ± 11.5 months; inclusion required >=5 years follow-up or death.	Prophylactic selective ND in 181/336. Of 155 initially observed, 40 developed clinical nodal relapse and underwent delayed modified radical ND.	No radiotherapy or chemotherapy before development of nodal metastasis; postoperative adjuvant details otherwise NR.	Nodal status established by histopathology after neck dissection or uneventful follow-up for at least 5 years.
Hori, 2017	Exact duration NR.	Observation after primary partial glossectomy; salvage neck dissection (ND) for neck recurrence.	NR	Linked Kanagawa cohort B; late neck recurrence was the surveillance endpoint.
Pedersen, 2017	Median follow-up 3.0 years (range 0.79-7.6) among patients alive at last evaluation.	All source-cohort patients underwent SNB; modeled N+ status also incorporated isolated subsequent nodal recurrence.	NR	Follow-up schedule NR; nodal endpoint was composite rather than pure SLNB positivity.
Sinha, 2018	24-168 months (mean 66 months).	Primary surgery; cervical management/surveillance NR.	NR	Primary full-text cohort n=65; locoregional recurrence and disease-related mortality were assessed over long-term follow-up.
Sakata, 2018	Median follow-up 53 months.	Transoral partial glossectomy alone; END excluded; postoperative observation for occult/delayed neck metastasis.	Neoadjuvant therapy excluded; postoperative adjuvant treatment details NR.	Occult neck metastasis developed in 16/97; exact surveillance schedule NR.
Shimizu, 2018	5-year DFS reported; exact follow-up duration NR.	Transoral resection; 12 patients underwent END and the remainder were observed. Short-interval imaging follow-up was recommended for high-risk invasive-front findings.	NR	Surveillance intensity was discussed clinically, but a uniform patient-level schedule was not extractable.
Yamanouchi, 2018	Median follow-up 11 months (range 1-42).	Glossectomy alone 19/23; concomitant ipsilateral SOHND 4/23. Among glossectomy-only patients, 10 developed secondary cervical LNM and 2 developed LRR + cervical LNM.	No adjuvant therapy before surgery; postoperative adjuvant treatment NR.	Small cohort with short follow-up; cervical events and DFS were both reported.
Ebihara, 2019	Median follow-up 43 months (range 6-121).	Wait-and-watch 42/64; END 22/64. In WW patients, 14 developed NLM, median 159 days (range 56-1074).	Adjuvant treatment details NR.	Mixed END/WW pathway; pN0 in WW defined by absence of subsequent neck metastasis at last observation.
Yamakawa, 2019	Exact duration NR.	Primary resection without END; wait-and-see strategy for delayed neck metastasis.	NR	Delayed neck metastasis was assessed during postoperative surveillance.
Dourado, 2020	Recurrence and survival follow-up reported; exact duration NR.	Primary surgery; cervical management/surveillance NR.	Postoperative radiotherapy/chemotherapy as indicated; regimen and early-stage subgroup treatment counts NR.	Only the separately extractable early-stage subgroup was used in the review.
Larson, 2020	2-year locoregional recurrence endpoint reported; exact overall follow-up duration NR.	Primary surgery; cervical management variable/NR.	NR	Follow-up was sufficient for 2-year recurrence analysis.

Report	Follow-up duration / timing	Postoperative / cervical surveillance and salvage strategy	Adjuvant treatment	Relevant notes
Parekh, 2020	DFS follow-up reported; exact duration NR.	Upfront resection with elective nodal dissection.	NR	Longitudinal surveillance schedule not reported.
Hori, 2020	Median follow-up 68 months (range 8–201); 5-y RC and DFS reported.	Primary surgery with longitudinal cervical surveillance; detailed visit/imaging schedule not fully specified.	NR	Linked Kanagawa cohort B; WPOI/BD were proposed to inform postoperative follow-up.
Hori, 2021	5-year RC/DFS framework; 15 neck recurrences occurred within 2 years; exact overall duration NR.	Primary tumor excision followed by surveillance; ND for neck recurrence.	NR	Linked Kanagawa cohort B; substantially overlaps Hori 2020.
Sakai, 2022	5-year RFS reported; exact follow-up duration NR.	END in 16 patients; no END in 54. Nodal metastasis was monitored during follow-up.	NR	Mixed cervical management precludes classification as a purely pathological or surveillance cohort.
Salzano, 2022	NR	Sentinel lymph node biopsy (SLNB) used for initial cervical assessment; subsequent surveillance schedule NR.	NR	Primary cervical endpoint was SLNB positivity.
Lakhera, 2023	NR	Transoral wide excision plus ipsilateral selective ND in all patients (systematic END); longitudinal surveillance schedule NR.	NR	Primary endpoint was pathological/occult nodal involvement after END. Radiotherapy was discussed as a possible management option for high-risk histology, not reported as a study treatment regimen.
Mohamed, 2023	NR	Definitive primary surgery; ND was not mandatory when the neck was clinically N0; follow-up schedule NR.	Postoperative radiotherapy (PORT) was included in multivariable survival analysis and was associated with OS; treatment counts/regimen NR.	Linked Cork cohort C; adjuvant-treatment benefit requires further study.
Beute, 2023	NR	Primary resection plus END.	NR	Primary endpoint was occult cervical metastasis on pathological assessment; long-term surveillance was not required for that endpoint.
Fonseca, 2023	Follow-up data available through January 2020; directly comparable duration NR.	Postoperative follow-up for locoregional recurrence; schedule NR.	NR	The report discussed selection for post-surgical adjuvant therapy but did not provide a regimen for the eligible cohort.
Kakuguchi, 2023	Median 73.2 months (range 7-207); mean time to regional neck metastasis 6 months (range 1-13).	Monthly during years 1-2; every 2-3 months in year 3; every 3-4 months in year 4; every 4-5 months in year 5; every 6 months thereafter. Contrast-enhanced head/neck CT every 3 months and chest CT every 6 months during the first 2 years; high-risk ultrasonography every 1-2 months; MRI/PET-CT when metastasis was suspected.	No chemotherapy or radiotherapy before cervical lymph-node metastasis (CLNM). Chemotherapy or radiotherapy was used in selected patients after CLNM/recurrence; numbers and indications NR.	Most detailed surveillance protocol among the included reports.
Yamauchi, 2023	5-year DSS reported; exact overall follow-up duration NR.	Primary resection without END; patients were followed until regional nodal recurrence, with salvage ND when recurrence was detected.	Postoperative treatment was used after salvage in recurrent cases; regimen NR.	Observation without initial END was central to the study design.
Yamazaki, 2023	Postoperative observation for >2 years; exact follow-up duration and surveillance schedule NR.	Partial glossectomy followed by postoperative observation; detailed surveillance schedule NR.	NR	Exact follow-up duration unavailable.
Dey, 2024	NR	All eligible patients underwent END; longitudinal surveillance schedule NR.	NR for the eligible early-stage cohort.	Primary endpoint was occult nodal metastasis/pathological nodal status.
Obayashi, 2024	NR	Primary surgery without END; postoperative surveillance for late cervical lymph-node metastasis; schedule and salvage strategy NR.	NR	Late nodal disease was the relevant cervical follow-up endpoint.
Singh, 2024	NR	Wide local excision plus neck dissection; longitudinal surveillance schedule NR.	NR	Primary endpoint was pathological lymph-node metastasis.
Zheng, 2024	LRRFS and OS analyzed; exact follow-up duration NR.	Primary surgery; cervical management/surveillance NR.	NR	Longitudinal outcomes were analyzed, but surveillance details were not reported.
Hintze, 2025	Mean 72 months; median 60 months.	END in 130 patients; remaining clinically N0 patients were observed. Salvage cervical surgery was used for regional	Among pN0 patients (n=98): no adjuvant treatment, n=59; PORT, n=36; postoperative	Linked Cork cohort C. PORT was specifically evaluated in relation to

Report	Follow-up duration / timing	Postoperative / cervical surveillance and salvage strategy	Adjuvant treatment	Relevant notes
		recurrence when feasible.	chemoradiotherapy, n=3.	regional recurrence.
Kikuchi, 2025	Overall follow-up duration NR; among late nodal metastases, median recurrence time 10 months (range 2–26).	Primary tumor resection; prophylactic ND at primary treatment in 8 patients; 13/17 patients with late nodal metastasis underwent ND as posterior/salvage therapy.	Neoadjuvant therapy was an exclusion criterion; postoperative adjuvant details NR.	Late nodal metastasis occurred in 17/66 (25.8%); detailed surveillance interval was not reported.
Alonso, 2026	Minimum postoperative follow-up 3 years by inclusion criterion; exact common median/mean follow-up not reported.	All 61 patients underwent extensive primary tumor resection plus elective neck dissection; DFS and OS were assessed longitudinally.	NR	Homogeneous cT1–2 cN0 oral-tongue cohort with negative surgical margins; Version of Record published online in 2025.
Goyal, 2026	NR	Pathological nodal status was recorded; exact cervical surgical procedure and longitudinal surveillance schedule were not explicitly reported.	NR	NR for long-term follow-up; exact cervical procedure remains genuinely unreported in the primary Methods.
Calabrese, 2026	NR	Primary surgery; END in 682/1109 patients, whereas 427/1109 were managed without END using clinical/ultrasound surveillance; detailed longitudinal surveillance schedule NR.	NR	Primary endpoint was occult nodal metastasis in a multicenter predictive-model cohort.

Abbreviations: CLNM, cervical lymph-node metastasis; CRT, chemoradiotherapy; DFS, disease-free survival; DSS, disease-specific survival; END, elective neck dissection; LRRFS, locoregional recurrence-free survival; MRI, magnetic resonance imaging; ND, neck dissection; NR, not reported or not directly verifiable in the primary report; OS, overall survival; PET-CT, positron-emission tomography/computed tomography; PORT, postoperative radiotherapy; RC, regional control; RFS, recurrence-free survival; SLNB, sentinel lymph node biopsy; US, ultrasonography.
Oncologic outcome data are reported separately in Tables S8-S10 and are not duplicated here. Follow-up and treatment details were retained only when they were explicitly reported or could be directly verified in the primary report; absence of reporting was not interpreted as absence of adjuvant treatment.
For reports with a synchronous pathological nodal endpoint after END or SLNB, a long-term follow-up interval was not required to ascertain the primary nodal outcome; these rows are nevertheless retained to document whether postoperative surveillance information was available.
Linked/partially overlapping reports (Almangush cohort A; Hori cohort B; Cork cohort C) are retained for non-redundant analyses but are not interpreted as independent patient cohorts.

Table S12. Final QUIPS risk-of-bias summary for the 38 included reports.

No.	Report	Study participation	Study attrition	Prognostic factor measurement	Outcome measurement	Study confounding	Statistical analysis and reporting
1	Li, 2013	Moderate	Moderate	Low	Moderate	Moderate	Low
2	Almangush, 2014	Moderate	Moderate	Low	Moderate	Moderate	Low
3	Almangush, 2015a	Moderate	Moderate	Moderate	Moderate	Moderate	Moderate
4	Almangush, 2015b	Moderate	Moderate	High	Moderate	Moderate	Low
5	Xie, 2015	Moderate	Moderate	High	High	Moderate	Moderate
6	Heerema, 2016	Moderate	Low	Low	Low	Moderate	Low
7	Arora, 2017	Moderate	Low	Moderate	Moderate	Moderate	Moderate
8	Hori, 2017	Moderate	Moderate	Low	Moderate	Moderate	Moderate
9	Pedersen, 2017	Moderate	Moderate	Low	Moderate	Moderate	Low
10	Sinha, 2018	Moderate	Moderate	Moderate	Moderate	High	Moderate
11	Sakata, 2018	Moderate	Moderate	Moderate	Moderate	Moderate	Moderate
12	Shimizu, 2018	Moderate	Moderate	Moderate	Moderate	Moderate	High
13	Yamanouchi, 2018	High	High	High	Moderate	High	High
14	Ebihara, 2019	Moderate	Moderate	High	High	Moderate	Moderate
15	Yamakawa, 2019	Moderate	Moderate	Low	Moderate	Low	Low
16	Dourado, 2020	High	Moderate	High	Moderate	High	Moderate
17	Larson, 2020	Moderate	Moderate	Moderate	Moderate	Moderate	Moderate
18	Parekh, 2020	Moderate	Moderate	Moderate	Low	Moderate	Low
19	Hori, 2020	Moderate	Low	Low	Low	Low	Low
20	Hori, 2021	Moderate	Moderate	Low	Low	Moderate	Moderate
21	Sakai, 2022	Moderate	Moderate	Moderate	Moderate	Low	Low
22	Salzano, 2022	Moderate	Low	Moderate	High	High	Low
23	Lakhera, 2023	Moderate	Low	Low	Moderate	Low	Low
24	Mohamed, 2023	Moderate	Moderate	Moderate	Moderate	Moderate	Low
25	Beute, 2023	Moderate	Low	Moderate	Low	Moderate	Low
26	Fonseca, 2023	High	Low	Moderate	Moderate	High	Moderate
27	Kakuguchi, 2023	Moderate	Low	High	Moderate	High	Low
28	Yamauchi, 2023	Moderate	Moderate	Low	Moderate	High	Moderate
29	Yamazaki, 2023	Moderate	Moderate	High	Moderate	Moderate	Moderate
30	Dey, 2024	Moderate	Low	Moderate	Moderate	Moderate	Low
31	Obayashi, 2024	Moderate	Moderate	Moderate	Moderate	Moderate	High
32	Singh, 2024	Low	Low	Moderate	Low	Low	Low
33	Zheng, 2024	Moderate	Moderate	High	Moderate	Low	Moderate
34	Hintze, 2025	High	High	High	Low	Moderate	Moderate
35	Kikuchi, 2025	Moderate	Moderate	Moderate	Moderate	Moderate	Moderate
36	Alonso, 2026	Moderate	Low	Moderate	Low	Moderate	Moderate
37	Goyal, 2026	Moderate	Moderate	Moderate	High	High	Moderate
38	Calabrese, 2026	Moderate	Low	High	Low	Low	Moderate

QUIPS = Quality In Prognosis Studies. Domains were judged as Low, Moderate, or High risk of bias.

For reports contributing more than one eligible outcome, the domain judgment reflects the more conservative appraisal across the outcomes synthesized in this review.

A missing or unverifiable methodological detail was not assumed to indicate low risk of bias. No numerical or global QUIPS score was calculated because QUIPS is a domain-based instrument.

Domain-level judgments reflected the overall pattern and likely impact of item-level concerns within each domain rather than a numerical summation. For prognostic-factor measurement, a prespecified conventional definition, blinded assessment, and independent or verified observer review supported lower-risk judgments, whereas study-specific composite definitions, selective reassessment procedures, absent blinding, or incompletely described measurement methods increased concern. This rule explains, for example, the High judgment for Hintze (expanded WPOI-5 construct; selective reassessment of cases with non-cohesive invasion/PNI/LVI; blinding not reported) versus the Low judgment for Lakhera (prespecified five-tier WPOI; two pathologists; blinded assessment with supplementary verification).

The 38-report QUIPS matrix below represents the final report-level assessment after primary-source reappraisal and the final eligibility audit. Linked or partially overlapping reports were appraised separately at report level, while cohort linkage was handled in the synthesis rather than treated as a QUIPS domain.

In the final QUIPS assessment, high-risk classifications were most frequent for prognostic-factor measurement (10/38) and study confounding (8/38), followed by outcome measurement (4/38), study participation (4/38), statistical analysis/reporting (3/38), and study attrition (2/38). No overall numerical QUIPS score was calculated.

Linked/partially overlapping reports (Almangush cohort A; Hori cohort B; Cork cohort C) were appraised at report level; linkage affects independence of evidence but was not itself treated as a QUIPS bias domain.
