# Supplementary material for: Prognostic Value of Tumor Budding and Worst Pattern of Invasion in Clinically Node-Negative Early-Stage Lip and Oral Cavity Squamous Cell Carcinoma: A Systematic Review
Source: J Clin Med. 2026 Oct 14;15(20):7949. doi: 10.3390/jcm15207949 (PMC13705793; doi:10.3390/jcm15207949)
Supplement: Supplementary file 1 [file jcm-15-07949-s001.zip › PRISMA 2020 Checklist JCM REPAGINATED.pdf]

PRISMA

PRISMA 2020 Checklist

Section and Topic	Item #	Checklist item	Location where item is reported
**TITLE**			
Title	1	Identify the report as a systematic review.	Title, p. 1.
**ABSTRACT**			
Abstract	2	See the PRISMA 2020 for Abstracts checklist.	Abstract, pp. 1–2.
**INTRODUCTION**			
Rationale	3	Describe the rationale for the review in the context of existing knowledge.	Introduction, pp. 2–3.
Objectives	4	Provide an explicit statement of the objective(s) or question(s) the review addresses.	Introduction, p. 3 (final paragraph: review objectives and eligible outcomes).
**METHODS**			
Eligibility criteria	5	Specify the inclusion and exclusion criteria for the review and how studies were grouped for the syntheses.	Methods, Section 2.3 (Eligibility Criteria), pp. 4-5; Section 2.6 (Outcomes), pp. 6; Section 2.8 (Data Synthesis), pp. 6-7.
Information sources	6	Specify all databases, registers, websites, organisations, reference lists and other sources searched or consulted to identify studies. Specify the date when each source was last searched or consulted.	Methods, Section 2.2 (Information Sources and Search Strategy), pp. 3–4; Supplementary Tables S1–S4, pp. 1–3. Source-specific verification dates are reported in Tables S1–S3.
Search strategy	7	Present the full search strategies for all databases, registers and websites, including any filters and limits used.	Supplementary Tables S1–S3, pp. 1–3 (full source-specific search strategies, limits, and verification dates); Table S4, p. 3 (final identification/deduplication accounting).
Selection process	8	Specify the methods used to decide whether a study met the inclusion criteria of the review, including how many reviewers screened each record and each report retrieved, whether they worked independently, and if applicable, details of automation tools used in the process.	Methods, Section 2.4 (Study Selection and Report Retrieval), p. 5. Following cross-source deduplication, all 1,831 unique records were independently screened at title/abstract level by two human reviewers, and the full texts of potentially eligible reports were subsequently assessed independently by the same two reviewers. Disagreements were resolved through discussion and, when necessary, consultation with a third reviewer. AI-assisted support (ChatGPT, OpenAI) was not used to pre-filter, rank, exclude, or determine the eligibility of records and did not generate a shortlist replacing human screening. AI assistance was limited to screening-record organization, bibliographic cross-checking, flagging potentially relevant or potentially inconsistent records for additional human review, and consistency checks across extracted data and manuscript tables. All final eligibility decisions were made by the human reviewers and verified against the original reports.
Data collection process	9	Specify the methods used to collect data from reports, including how many reviewers collected data from each report, whether they worked independently, any processes for obtaining or confirming data from study investigators, and if applicable, details of automation tools used in the process.	Methods, Sections 2.4–2.5, pp. 5–6. Data were extracted by one reviewer and independently verified by a second reviewer. Study-level data entering the synthesis were checked against primary reports; when a numerical effect estimate could not be verified directly, the association was summarized qualitatively and no numerical estimate from a secondary extraction was substituted.

PRISMA 2020 Checklist

Section and Topic	Item #	Checklist item	Location where item is reported
Data items	10a	List and define all outcomes for which data were sought. Specify whether all results that were compatible with each outcome domain in each study were sought (e.g. for all measures, time points, analyses), and if not, the methods used to decide which results to collect.	Methods, Section 2.6 (Outcomes), pp. 6; Supplementary Tables S8–S10, pp. 12–19. Nodal, recurrence, and survival outcomes are defined and kept clinically distinct.
	10b	List and define all other variables for which data were sought (e.g. participant and intervention characteristics, funding sources). Describe any assumptions made about any missing or unclear information.	Methods, Section 2.5, p. 5-6; Supplementary Table S5, pp. 4–6; Table S7, pp. 8–11; Table S10, pp. 18–19; Table S11, pp. 20–22. Variables include study/population, staging/TNM edition, cervical management, TB/WPOI methods, histopathology, follow-up, and adjuvant treatment. Missing or unverifiable details are reported as NR rather than inferred; study-level numerical estimates retained in the synthesis were checked against primary reports.
Study risk of bias assessment	11	Specify the methods used to assess risk of bias in the included studies, including details of the tool(s) used, how many reviewers assessed each study and whether they worked independently, and if applicable, details of automation tools used in the process.	Methods, Section 2.7 (Risk-of-Bias Assessment), p. 6; Supplementary Table S12, p. 23. QUIPS assessments were performed independently by two reviewers, with disagreements resolved by discussion and, when necessary, consultation with a third reviewer. Domain-level judgments reflected the overall pattern and likely impact of item-level concerns rather than a numerical score; missing or unclear information was not assumed to indicate low risk. No automated tool assigned QUIPS judgments.
Effect measures	12	Specify for each outcome the effect measure(s) (e.g. risk ratio, mean difference) used in the synthesis or presentation of results.	Methods, Section 2.5, p. 5-6. ORs, RRs, and HRs with 95% CIs were extracted when available; univariable and adjusted estimates were distinguished; reported time-to-event estimates were preferred to newly calculated crude effects.
Synthesis methods	13a	Describe the processes used to decide which studies were eligible for each synthesis (e.g. tabulating the study intervention characteristics and comparing against the planned groups for each synthesis (item #5)).	Methods, Sections 2.6 and 2.8, pp. 6–7. Studies were grouped by prognostic marker and clinically distinct outcome; linked/overlapping cohorts were handled as related evidence.
	13b	Describe any methods required to prepare the data for presentation or synthesis, such as handling of missing summary statistics, or data conversions.	Methods, Sections 2.5 and 2.8, pp. 6–7. Primary-reported estimates were retained; no post hoc conversion of time-to-event estimates was undertaken; overlapping participants were not double counted.
	13c	Describe any methods used to tabulate or visually display results of individual studies and syntheses.	Methods, Section 2.8, pp. 6–7; Supplementary Tables S5–S12, pp. 4–23. Results are presented in structured report-level tables and outcome-specific narrative synthesis.
	13d	Describe any methods used to synthesize results and provide a rationale for the choice(s). If meta-analysis was performed, describe the model(s), method(s) to identify the presence and extent of statistical heterogeneity, and software package(s) used.	Methods, Section 2.8 (Data Synthesis), pp. 6–7. Narrative synthesis was selected because heterogeneity involved not only effect magnitude but also the definition of the prognostic exposures and clinical endpoints. TB/WPOI definitions and thresholds, cervical-management pathways, outcome ascertainment, TNM edition, follow-up, effect measures, covariate adjustment, and cohort independence varied substantially, such that a single

PRISMA 2020 Checklist

Section and Topic	Item #	Checklist item	Location where item is reported
			pooled estimate was considered of uncertain clinical meaning. A restricted subgroup meta-analysis was also considered for studies using the common <5- versus ≥5-bud TB threshold; however, residual differences in staining/counting methods, field definition, cervical management, outcome ascertainment, and adjustment and the limited number of sufficiently comparable independent cohorts within individual outcomes precluded a meaningful pooled estimate. Findings were therefore synthesized narratively by prognostic marker, outcome, and cervical-management pathway.
	13e	Describe any methods used to explore possible causes of heterogeneity among study results (e.g. subgroup analysis, meta-regression).	Methods, Section 2.8 (Data Synthesis), pp. 6–7; Results, pp. 7–14; Discussion, pp. 14–17. Possible sources of heterogeneity were explored descriptively according to prognostic-marker definition and threshold, histopathological assessment method, cervical-management pathway, outcome ascertainment, TNM edition, statistical adjustment, and cohort independence. A restricted subgroup meta-analysis was specifically considered for studies using the common <5- versus ≥5-bud TB threshold; however, residual methodological and clinical heterogeneity and the limited number of sufficiently comparable independent cohorts within individual outcomes precluded a meaningful pooled subgroup estimate. No formal meta-regression was performed.
	13f	Describe any sensitivity analyses conducted to assess robustness of the synthesized results.	Not performed. No pooled meta-analysis was undertaken, and no formal sensitivity analysis was conducted.
Reporting bias assessment	14	Describe any methods used to assess risk of bias due to missing results in a synthesis (arising from reporting biases).	Not formally assessed. No formal reporting-bias/publication-bias analysis was performed because the evidence was synthesized narratively across heterogeneous outcomes and effect measures.
Certainty assessment	15	Describe any methods used to assess certainty (or confidence) in the body of evidence for an outcome.	Not formally assessed. No GRADE or other certainty-of-evidence framework was applied.
**RESULTS**			
Study selection	16a	Describe the results of the search and selection process, from the number of records identified in the search to the number of studies included in the review, ideally using a flow diagram.	Results, Section 3.1 (Study Selection), p. 7; Figure 1, p. 8; Supplementary Table S4, p. 3.
	16b	Cite studies that might appear to meet the inclusion criteria, but which were excluded, and explain why they were excluded.	Methods, Section 2.3, pp. 4–5 (eligibility and exclusion criteria); Results, Section 3.1, p. 7. Full-text exclusions are reported in aggregate (n = 254). Individual near-eligible excluded reports are not separately cited in the final manuscript; therefore, PRISMA item 16b is only partially addressed.
Study characteristics	17	Cite each included study and present its characteristics.	Results, Section 3.2 (Characteristics of the Included Evidence), p. 8; Supplementary Table S5, pp. 4–6. All 38 included reports are cited in the manuscript [32–69] and characterized at report level.

PRISMA 2020 Checklist

Section and Topic	Item #	Checklist item	Location where item is reported
Risk of bias in studies	18	Present assessments of risk of bias for each included study.	Results, Section 3.8 (Risk of Bias), p. 14; Supplementary Table S12, p. 23 (report-level judgments across all six QUIPS domains).
Results of individual studies	19	For all outcomes, present, for each study: (a) summary statistics for each group (where appropriate) and (b) an effect estimate and its precision (e.g. confidence/credible interval), ideally using structured tables or plots.	Results, pp. 7–14; Supplementary Tables S8–S10, pp. 12–19. Study-specific event data, effect estimates, 95% CIs, and adjusted/unadjusted analyses are reported where available.
Results of syntheses	20a	For each synthesis, briefly summarise the characteristics and risk of bias among contributing studies.	Results, pp. 7–14; Supplementary Table S5, pp. 4–6, and Table S12, p. 23. Study characteristics, linked cohorts, and risk-of-bias patterns are summarized alongside the outcome syntheses.
	20b	Present results of all statistical syntheses conducted. If meta-analysis was done, present for each the summary estimate and its precision (e.g. confidence/credible interval) and measures of statistical heterogeneity. If comparing groups, describe the direction of the effect.	No pooled statistical synthesis was conducted. Outcome-specific narrative syntheses and study-specific effect estimates are presented in Results, pp. 7–14, and Supplementary Tables S8–S10, pp. 12–19.
	20c	Present results of all investigations of possible causes of heterogeneity among study results.	Results, pp. 7–14; Discussion, pp. 14–17. Sources of heterogeneity are described by WPOI/TB definition, threshold, cervical-management pathway, endpoint definition, follow-up, and adjustment set; no formal heterogeneity model was performed.
	20d	Present results of all sensitivity analyses conducted to assess the robustness of the synthesized results.	Not performed; no formal sensitivity analyses were conducted.
Reporting biases	21	Present assessments of risk of bias due to missing results (arising from reporting biases) for each synthesis assessed.	Not formally assessed; no formal reporting-bias analysis was conducted.
Certainty of evidence	22	Present assessments of certainty (or confidence) in the body of evidence for each outcome assessed.	Not formally assessed; no formal certainty-of-evidence rating was conducted.
**DISCUSSION**			
Discussion	23a	Provide a general interpretation of the results in the context of other evidence.	Discussion, pp. 14–17. Findings are interpreted in relation to previous systematic reviews/meta-analyses and the expanded early-stage evidence base.
	23b	Discuss any limitations of the evidence included in the review.	Discussion, pp. 16–17 (limitations of the evidence: linked cohorts, retrospective designs, heterogeneous definitions/outcomes/follow-up/statistical methods, incomplete reporting, and subsite imbalance).
	23c	Discuss any limitations of the review processes used.	Discussion, Limitations, pp. 16–17. Review-process limitations include the absence of prospective registration and a prespecified protocol, omission of Embase from the bibliographic database search, restriction to English-language full-text articles, and the fixed literature eligibility cutoff of 25 April 2026. Methods, Sections 2.1–2.8, pp. 4–9, describe the review procedures and search-verification process.
	23d	Discuss implications of the results for practice, policy, and future research.	Discussion, pp. 14–17; Conclusions, pp. 17–18. Clinical implications are deliberately cautious; TB/WPOI are presented as complementary postoperative markers and future prospective multicenter validation is recommended.
**OTHER INFORMATION**			

PRISMA 2020 Checklist

Section and Topic	Item #	Checklist item	Location where item is reported
Registration and protocol	24a	Provide registration information for the review, including register name and registration number, or state that the review was not registered.	Methods, Section 2.1 (Study Design and Reporting), p. 3. The review was not prospectively registered in PROSPERO; the absence of prospective registration is acknowledged as a methodological limitation.
	24b	Indicate where the review protocol can be accessed, or state that a protocol was not prepared.	Methods, Section 2.1 (Study Design and Reporting), p. 3. The review was not prospectively registered in PROSPERO, and no separate review protocol document was prepared.
	24c	Describe and explain any amendments to information provided at registration or in the protocol.	No registered protocol was available to amend. Search-verification changes are transparently described in Methods, Section 2.2, pp. 3–4, and Supplementary Tables S1–S4, pp. 1–3.
Support	25	Describe sources of financial or non-financial support for the review, and the role of the funders or sponsors in the review.	Funding statement, p. 18: "This research received no external funding."
Competing interests	26	Declare any competing interests of review authors.	Conflicts of Interest statement, p. 19: The authors declare no conflicts of interest.
Availability of data, code and other materials	27	Report which of the following are publicly available and where they can be found: template data collection forms; data extracted from included studies; data used for all analyses; analytic code; any other materials used in the review.	Data Availability Statement, p. 18; Supplementary Tables S1–S12, pp. 1–23. Data supporting the review are provided in the article and Supplementary Materials.

Completed for the final revised manuscript (38 included reports; 254 full-text exclusions).

From: Page MJ, McKenzie JE, Bossuyt PM, Boutron I, Hoffmann TC, Mulrow CD, et al. The PRISMA 2020 statement: an updated guideline for reporting systematic reviews. BMJ 2021;372:n71. doi: 10.1136/bmj.n71.
This work is licensed under CC BY 4.0. To view a copy of this license, visit https://creativecommons.org/licenses/by/4.0/
